# Luminescent platinum(ii) complexes with self-assembly and anti-cancer properties: hydrogel, pH dependent emission color and sustained-release properties under physiological conditions†
†Electronic supplementary information (ESI) available: Synthesis and characterization data, biological studies, supplementary figures/tables. X-ray crystallographic data of **1**(ClO_4_). CCDC 1058788. For ESI and crystallographic data in CIF or other electronic format see DOI: 10.1039/c4sc03635b


**DOI:** 10.1039/c4sc03635b

**Published:** 2015-04-28

**Authors:** Johnson Lui-Lui Tsai, Taotao Zou, Jia Liu, Tianfeng Chen, Anna On-Yee Chan, Chen Yang, Chun-Nam Lok, Chi-Ming Che

**Affiliations:** a State Key Laboratory of Synthetic Chemistry , Institute of Molecular Functional Materials , Chemical Biology Centre and Department of Chemistry , The University of Hong Kong , Pokfulam Road , Hong Kong , China . Email: cmche@hku.hk; b HKU Shenzhen Institute of Research and Innovation , Shenzhen 518053 , China; c Department of Chemistry , Jinan University , Guangzhou 510632 , China

## Abstract

Supramolecular interactions are of paramount importance in biology and chemistry, and can be used to develop new vehicles for drug delivery. Recently, there is a surge of interest on self-assembled functional supramolecular structures driven by intermolecular metal–metal interactions in cellular conditions. Herein we report a series of luminescent Pt(ii) complexes [Pt(C^N^N^pyr^)(C≡NR)]^+^ [HC^N^N^pyr^ = 2-phenyl-6-(1*H*-pyrazol-3-yl)-pyridine)] containing pincer type ligands having pyrazole moieties. These Pt(ii) complexes exert potent cytotoxicity to a panel of cancer cell lines including primary bladder cancer cells and display strong phosphorescence that is highly sensitive to the local environment. The self-assembly of these complexes is significantly affected by pH of the solution medium. Based on TEM, SEM, ESI-MS, absorption and emission spectroscopy, and fluorescence microscopy together with cell based assays, [Pt(C^N^N^pyr^)(C≡NR)]^+^ complexes were observed to self-assemble into orange phosphorescent polymeric aggregates driven by intermolecular Pt(ii)–Pt(ii) and ligand–ligand interactions in a low-pH physiological medium. Importantly, the intracellular assembly and dis-assembly of [Pt(C^N^N^pyr^)(C≡NR)]^+^ are accompanied by change of emission color from orange to green. These [Pt(C^N^N^pyr^)(C≡NR)]^+^ complexes accumulated in the lysosomes of cancer cells, increased the lysosomal membrane permeability and induced cell death. One of these platinum(ii) complexes formed hydrogels which displayed pH-responsive and sustained release properties, leading to low-pH-stimulated and time-dependent cytotoxicity towards cancer cells. These hydrogels can function as vehicles to deliver anti-cancer agent cargo, such as the bioactive natural products studied in this work.

## Abstract 2

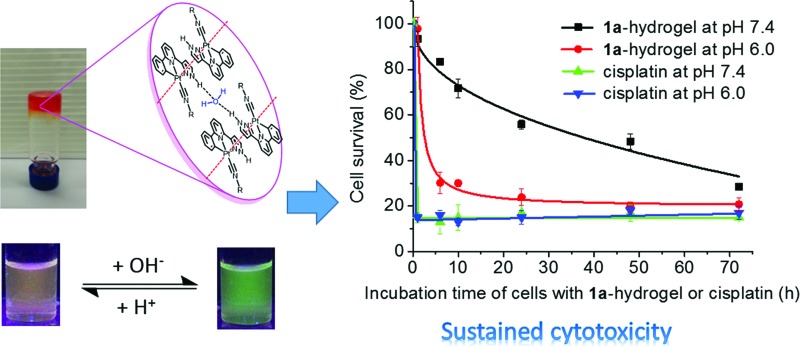
Luminescent platinum(ii) complexes show anti-cancer and pH-dependent self-assembly and sustained-release properties under physiological conditions.

## Introduction

Self-assembly of π-conjugated organometallic complexes driven by non-covalent interactions, such as metal–ligand coordination, metal–metal and ligand–ligand interactions, has spurred extensive studies by virtue of the intriguing optical, electronic and chemical properties resulting from these interactions.^1^ While supramolecular metal complexes, including those of gold, silver, copper, palladium, and platinum have been actively studied in materials science and catalysis,^2^ their therapeutic potential remains under-developed.^3^,^4^ Over the years, we have studied the synthesis and photophysical properties of luminescent organoplatinum(ii) complexes and their applications in optical devices, luminescent bio-sensors and medicines.^5^ We had reported a platinum(ii) complex, [Pt(C^N^N)(C≡NR^1^)]Cl (HC^N^N = 6-phenyl-2,2′-bipyridine, R^1^ = 2,6-dimethylphenyl) which can form chromonic mesophases/viscous fluids *via* d^8^–d^8^ metallophilic and ligand π–π interactions.5*d* This [Pt(C^N^N)(C≡NR^1^)]Cl complex undergoes aggregation in cancer cells, showing persistent ^3^MMLCT (triplet metal–metal to ligand charge transfer) emission in the cytoplasm.^6^

There has been a growing interest in developing metal complexes which target DNA as well as proteins of relevance to cancers.^7^ Such metal complexes offer new opportunity in the search of new anti-cancer metal medicines though resolution of toxic side effect of metal complexes has always been a challenge in this field.^8^ Nano-formulation has been shown to be able to improve the stability, solubility and selectivity of anti-cancer drug candidates and hence emerges as an appealing strategy to reduce toxic side effects.^6^,8b,c,^9^ In this context, we have reported the use of an organogel formed by [Au^III^(C^N^C)(4-dpt)]OTf [HC^N^CH = 2,6-diphenylpyridine; 4-dpt = 2,4-diamino-6-(4-pyridyl)-1,3,5-triazine], which displayed anti-proliferative and sustained release properties in biological systems.^10^ Nonetheless, the relatively toxic acetonitrile used in the organogel formation is undesirable from the perspective of clinical applications.

**Scheme 1:**
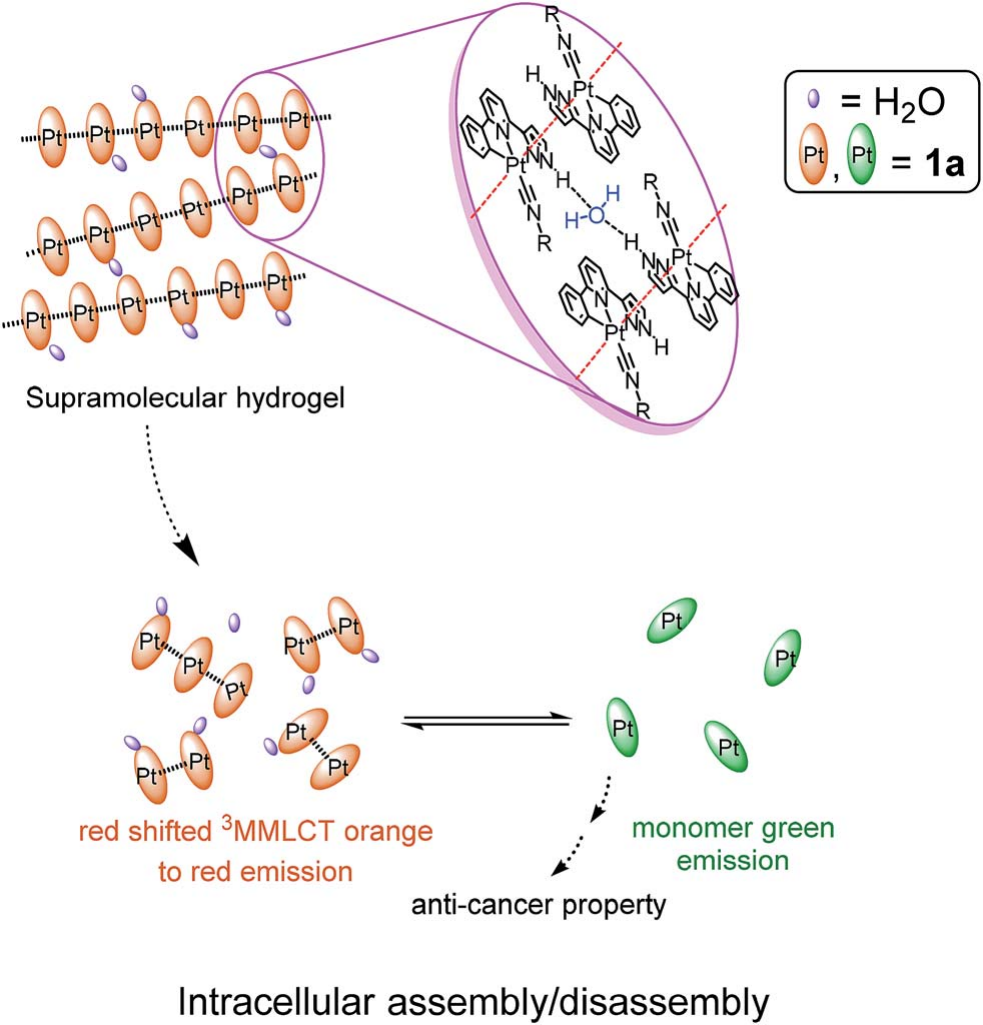
Induction of cancer cell death by release properties of supramolecular hydrogels.

**Fig. 1:**
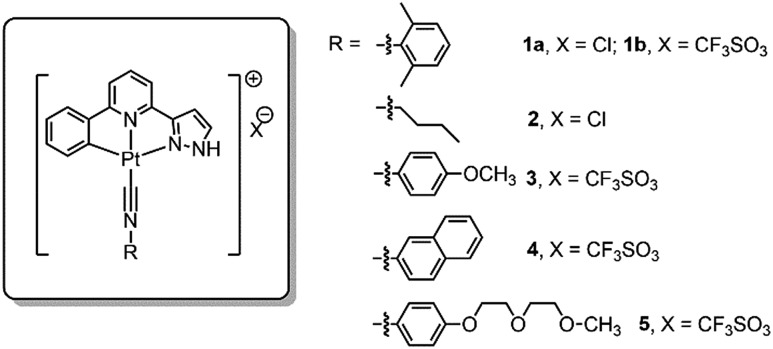
Chemical structures of the platinum(ii) complexes.

Pincer type platinum(ii) complexes are well documented to have rich luminescent properties, and more recently, to show anti-cancer properties. Indeed, examples of pincer type platinum complexes which display promising *in vivo* anti-tumor effect in animal model studies have recently been disclosed.7i,^11^ For these reasons, we initiated a program aiming to develop hydrogels with anti-cancer active Pt(ii) complexes (Scheme 1). Platinum(ii) complexes which form poly-electrolytes having hydrogel properties have been reported, an example of which is the dinuclear complex having [Pt(C^N^N)(C≡NR^1^)]^+^ motifs covalently connected to oligo(oxyethylene) chains.^12^ Formation of hydrogels by using H-bonding motifs has been reported in the literature.4b,^13^ Herein is described a series of phosphorescent platinum(ii) isocyanide complexes containing the C-deprotonated C^N^N^pyr^ ligand (HC^N^N^pyr^ = 2-phenyl-6-(1H-pyrazol-3-yl)-pyridine) (Fig. 1). Compared to [Pt(C^N^N)(C≡NR^1^)]Cl, [Pt(C^N^N^pyr^)(C≡NR^1^)]Cl, **1a** (Fig. 1), displays the following features: (1) it can form a supramolecular gel in water (denoted as **1a**-hydrogel); (2) its self-aggregation and emission properties in aqueous solutions are pH-dependent; (3) it selectively forms aggregates in low-pH lysosomes in cancer cells; the accumulation of Pt(ii) complexes in lysosomes is suggested to contribute to its cytotoxicity towards cancer cells; (4) **1a**-hydrogel exhibits sustained-release cytotoxicity towards cancer cells. Notably, the release activity of **1a**-hydrogel can be stimulated by acidic environment; (5) **1a**-hydrogel can encapsulate and deliver bioactive natural products, an example of which is the anti-metastatic, berberine.

## Results and discussion

### Synthesis and photophysical properties of the Pt(ii) complexes

The cyclometalated platinum(ii) complexes containing various isocyanide ligands (Fig. 1) were prepared by the reaction of [Pt(C^N^N^pyr^)Cl] with excess isocyanide ligand at room temperature for 12 h, and were characterized by ^1^H NMR spectroscopy, ESI-MS and elemental analyses (see details in ESI†). The ^1^H-^1^H COSY and ^1^H-^1^H NOESY NMR spectra of **1a** in d_4_-MeOH are shown in Fig. S1.† Their UV/Vis absorption and emission spectra, measured in CH_2_Cl_2_, CH_3_CN, and MeOH at 298 K, are given in the ESI (Tables S1 and S2 and Fig. S2 and S3†). Complexes **1–5** display similar absorption spectra and emission spectra. The absorption bands at 260–375 nm are attributed to the intraligand (^1^IL, π(L) → π*(L)) transitions of the C^N^N^pyr^ ligand while the lower energy absorption beyond 375 nm can be assigned to singlet dπ(Pt) → π*(L) metal-to-ligand charge transfer (^1^MLCT) transition.^14^ Upon light excitation, these complexes display vibronic structured emission with *λ*_max_ at 503–507 nm and lifetimes in the range of 7.4–15.6 μs, which are attributed to triplet excited states with mixed ^3^IL and ^3^MLCT character.

### Self-assembly of complex **1a** in water and formation of **1a**-hydrogel

**Fig. 2:**
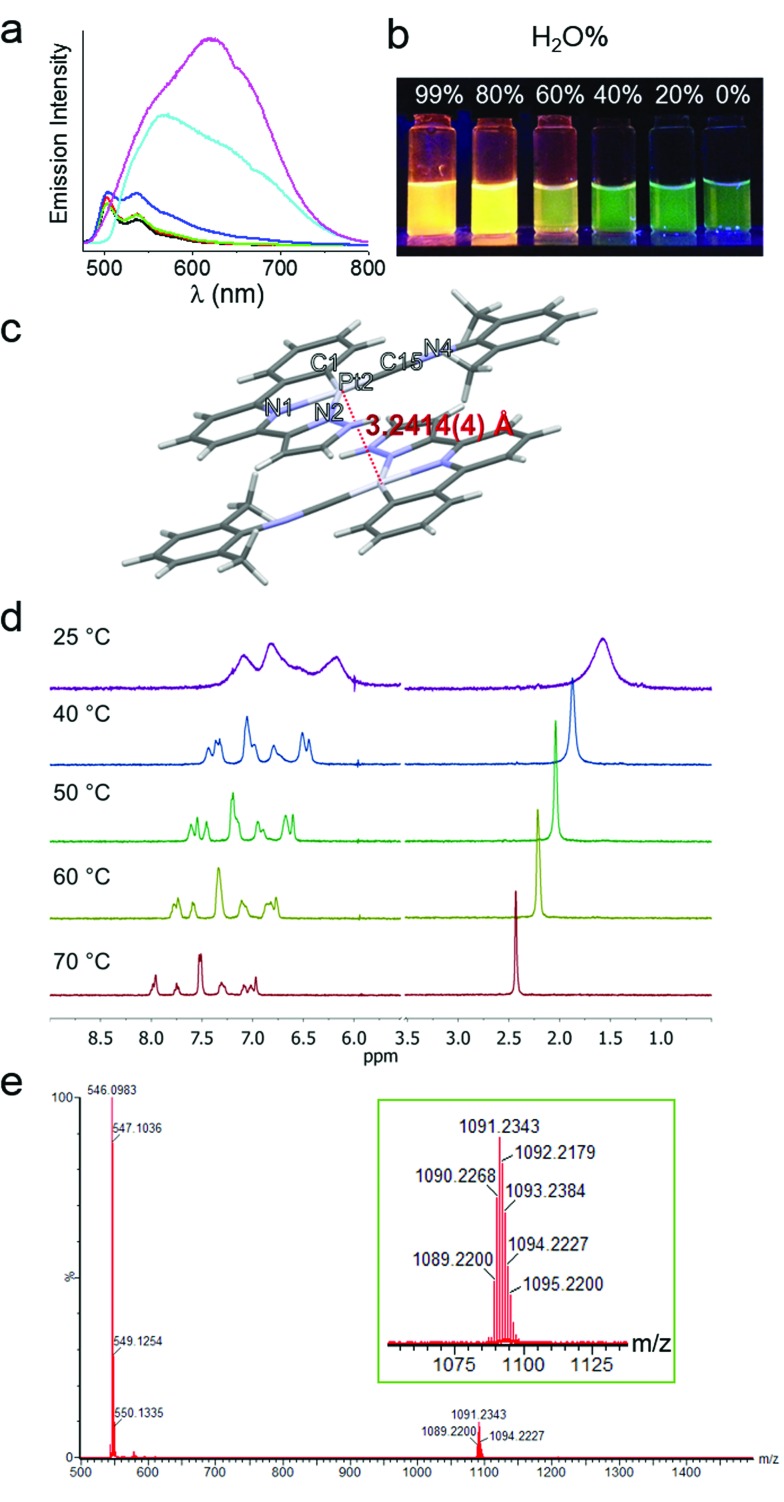
(a) Emission spectra of **1a** (20 μM) in solutions of varied MeOH : H_2_O ratio. (b) Sample images under UV irradiation at 365 nm. (c) Crystal structure of **1**(ClO_4_) displaying Pt–Pt interactions. (d) ^1^H NMR of **1a** (1 mM) in D_2_O with variation of temperatures. (e) ESI-MS spectrum of **1a** in 1% MeOH in H_2_O; inset shows *m*/*z* at a range of 1075–1125.

The spectroscopic properties of **1a** in aerated aqueous solutions having different H_2_O : MeOH ratios (1–99% H_2_O in MeOH, v/v) were examined (Fig. 2a). Complex **1a** displays green emission (*λ*_max_ = 503 nm) in MeOH. Upon increasing the H_2_O content, the emission color gradually changes to yellow-orange (Fig. 2b). In 80–99% H_2_O solution, the 503 nm green emission is replaced by a more intense and red-shifted broad emission with *λ*_max_ = ∼630 nm for the solution having 99% H_2_O (Fig. 2a); this low energy emission band is attributed to ^3^MMLCT [dσ*(Pt–Pt) → π*(C^N^N)] excited state. Parallel study using UV/Vis absorption spectroscopy also revealed the appearance of a new absorption band at ∼375 nm, concomitant with the decrease of absorbance at ∼350 nm (Fig. S4†). The crystal structure of **1a** analogue having ClO_4_^–^ as anion (Fig. 2c and Table S3†) displays Pt–Pt distance of 3.2414(4) Å, suggestive of Pt–Pt interactions. Temperature dependent (298–343 K) ^1^H NMR spectra of **1a** (1 mM in D_2_O) were measured (Fig. 2d). At 298 K, the signals in the aromatic region are broad and poorly resolved, indicative of aggregation. Upon increasing the temperature, the broad peaks are resolved into sharp signals and downfield shifted, implying the disassembly of **1a**-aggregates. The ESI-MS spectrum of **1a** in water (containing 1% MeOH; Fig. 2e) reveals the presence of the [Pt(C^N^N^pyr^)C≡NR^1^]^+^ cation at *m*/*z* 546.1 together with a singly charged cation at *m*/*z* 1091.2 attributed to the dimeric species (2[M]^+^ – H). All these findings are congruent with the formation of Pt(ii) aggregates in water, reminiscent of the previously reported self-aggregation behavior of [Pt(C^N^N)(C≡NR^1^)]Cl.

**Fig. 3:**
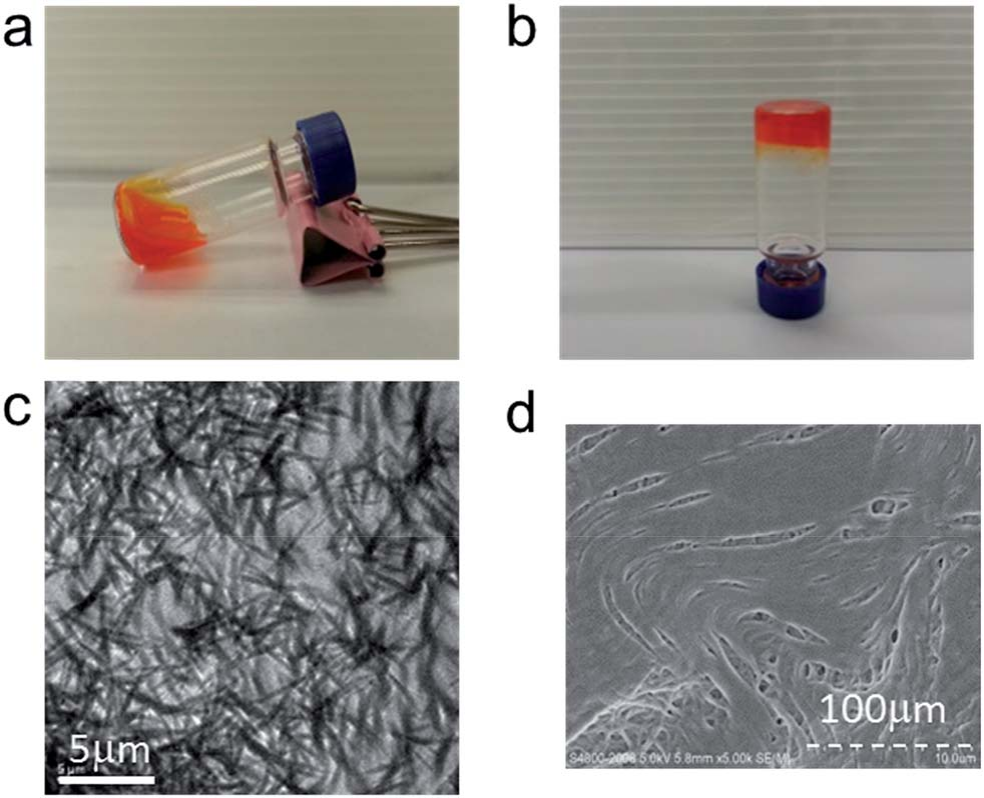
(a) Dissolution of **1a** (5.0 wt%) after heating at 80 °C. (b) Formation of hydrogels after cooling the solution to room temperature (right). (c) TEM and (d) SEM images of the viscous solution of **1a**.

We examined whether **1a** could form hydrogels since the pyrazole moiety supports an intermolecular H-bonding interaction. Upon heating a suspension of **1a** (5.0 wt%, ∼86 mM) in water to 80 °C, the complex completely dissolved to give a clear solution (Fig. 3a); after cooling to 25 °C, a viscous orange solution was formed (Fig. 3b). This process was reversible. Examination by transmission electron microscopy (TEM; Fig. 3c) and scanning electron microscopy (SEM; Fig. 3d) of the viscous solution of **1a** revealed partially aligned nano-fibers with diameters and lengths of hundreds of nanometers and several micrometers, respectively, which could account for the high viscosity of **1a** at this concentration.

**Fig. 4:**
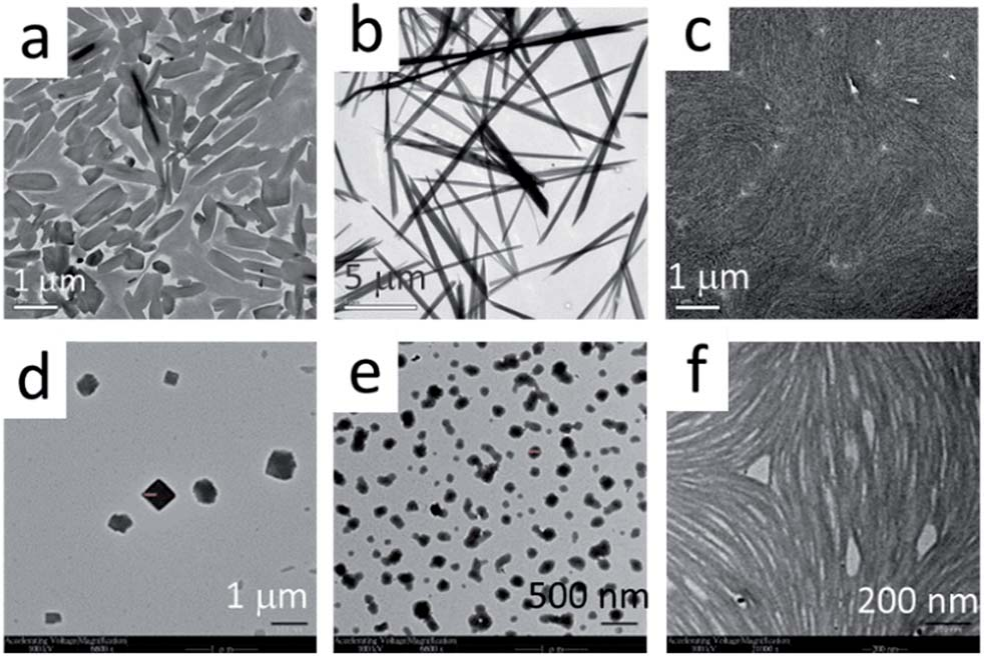
TEM images of complexes **1a** (a), **1b** (b), **2** (c), **3** (d), **4** (e), and **5** (f). The samples were prepared from evaporation of EtOH solutions of each complex.

TEM and SEM analyses further revealed that the Pt(ii) complexes having different isocyanide ligands, **1b** and **2–5**, could form different nano-aggregates, including nanorods (**1b**), nanocubes (**2**), nanospheres (**3**), and nanofibers (**4**,**5**) by evaporation of their ethanol solutions (Fig. 4 and S5†). However, no hydrogels from these complexes were observed.

### Self-assembly of the Pt(ii) complexes in low pH medium and in the acidic lysosomes of cancer cells

**Fig. 5:**
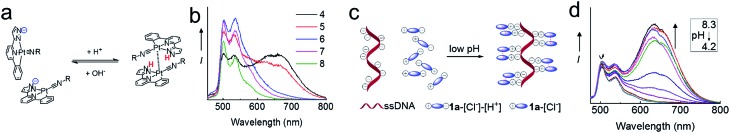
(a) Low pH-induced assembly (Pt–Pt interactions) of the Pt(ii) complex. (b) The emission spectra of **2** in different pH. (c) Assembly of cationic Pt(ii) complexes in the phosphate backbone of ssDNA. (d) The emission spectra of **1a** in the presence of ssDNA at pH of 8.3 to 4.2.

The NH unit of pyrazole is pH-sensitive (R_2_NH ↔ R_2_N^–^). Lam, Wong and coworkers reported that low pH (favoring the R_2_NH form) facilitates intramolecular Pt–Pt interactions in dinuclear [Pt_2_(C^N^N^pyr^)_2_(μ-dppm)]^2+^ while high pH (favoring R_2_N^–^) does not.^15^ By using emission spectroscopy, we examined the effect of pH on the intermolecular Pt–Pt interactions of the [Pt(C^N^N^pyr^)isocyanide]^+^ complexes in phosphate buffered saline solutions having different pHs ([phosphate] = 4 mM, the pH was adjusted by NaOH or HCl, final [NaCl] = 155 mM). Taking **2** as an example, at pH 8, the complex in a mixture of buffer/acetonitrile (4/1, v/v) showed a vibronic structured emission (*λ*_max_ = 504 nm) which is typical of the emission of mononuclear [Pt(C^N^N^pyr^)isocyanide]^+^ complexes (Fig. 5b). Upon lowering of the pH to 7 and 6, the emission intensity at *λ*_max_ = 504 nm increased; further decreasing the pH to 5 and 4 resulted in a broad emission band at *λ* = 670 nm that could be attributed to a ^3^MMLCT excited state. The increase in ^3^MMLCT emission was accompanied by a concomitant decrease in emission intensity at *λ* = 504 nm. That is, **2** is present in both monomeric and aggregated forms at pH 4 and 5. Dynamic light scattering experiments also indicated formation of aggregates of **2** with diameters of over hundreds of nanometers at pH of 4 (Fig. S6†). All other Pt(ii) complexes were observed to form aggregates displaying red-shifted emissions at low pH (Fig. S7†).

We next examined the pH-dependent emission properties of **1a** in the buffer solution/CH_3_CN (3/1, v/v) containing single stranded DNA (ssDNA, dA_20_). The anionic phosphate backbone in ssDNA is known to facilitate assembly of cationic Pt(ii) complexes.^16^ As shown in Fig. 5d and S8,† **1a** shows a broad low-energy emission peaked at ∼640 nm at pH 4 in the presence of ssDNA; however, there is no significant low-energy red emission in the absence of ssDNA or in the presence of peptide nucleic acid (PNA) which does not contain the anionic backbone. The broad prominent peak at ∼640 nm observed at low pH is indicative of the self-assembly of the positively charged **1a** ion on the nucleic acid backbone. Upon an increase in pH, the intensity of the long wavelength emission band decreases, which can be accounted for by the disassembling of surface bound Pt(ii) aggregates. It is conceivable that at high pH, deprotonation of pyrazole-H weakens the ionic binding interactions between the surface bound Pt(ii) complex and anionic ssDNA backbone, thus disrupting the intermolecular Pt–Pt interactions along the surface of the DNA backbone. In contrast, the cationic form of the Pt complex in low pH undergoes self-assembly, which is synergistically enhanced by the ionic interactions between the cationic Pt complex and anionic DNA (Fig. 5c). Complex **1b** also displays similar changes in emission properties at different pH (Fig. S9†).

**Fig. 6:**
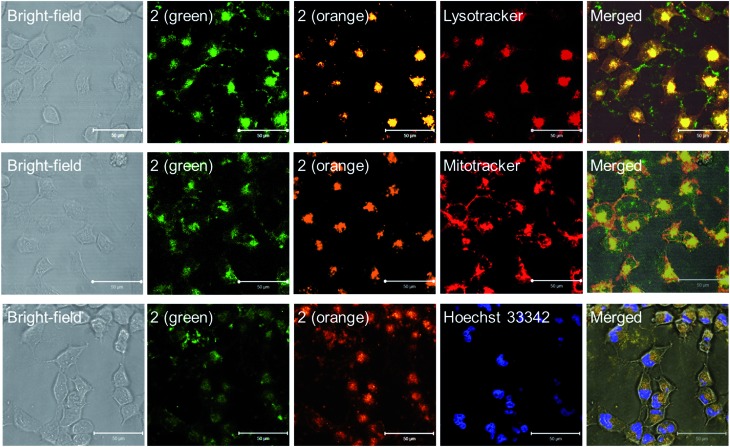
Fluorescence microscopic analyses of **2** (10 μM) in HeLa cells co-incubated with Lysotracker® Red DND-99 (50 nM, *E*_x_: 543 nm; *E*_m_: 565–615 nm) (top), Mitotracker (50 nM, *E*_x_: 543 nm; *E*_m_: 565–615 nm) (middle) or Hoechst 33342 (1 μM *E*_x_: 800 nm [two photon]; *E*_m_: 435–485 nm) (bottom). The **2** (green) and **2** (orange) are both excited at 458 nm but with emission channels of 500–550 nm and 650–700 nm, respectively.

Lysosomes are acidic cellular compartments (pH = ∼5) harboring acid hydrolases for the degradation of obsolete biomolecules and unwanted materials. We tested whether the Pt(ii) complexes accumulate in acidic lysosomes by fluorescence microscopy. After treatment of HeLa cells (human cervical carcinoma) with **1a** or **2** (10 μM) for 20 min, green emission could be detected in the cytoplasm (Fig. S10†), indicating efficient cellular uptake. Yellow-orange vesicles also appeared and gradually became predominant after 1–2 h. The specific cellular location of **2** after 1 h treatment was further examined by confocal microscopy (Fig. 6 and S11†). Upon excitation at 458 nm, in emission channel of 500–550 nm, the green emission was found to mainly localize in cytoplasmic structures and the cell membrane; while in the 650–700 nm channel, the orange/red emission was found to be specifically localized in vesicles. Co-staining of **2**-treated HeLa cells with LysoTracker® Red revealed an overlap of the orange and green emission with the LysoTracker® red signal, showing Pearson's coefficient *R* = 0.75 and 0.60, respectively. Both the green and orange emission of **2** did not exhibit co-localization with a specific stain for mitochondria (Mitotracker® Red) or nuclei (Hoechst 33342) (*R* < 0.15). The existence of both green and orange emission in low-pH lysosomes is consistent with the aforementioned emission measurements in solutions of different pH.

**Fig. 7:**
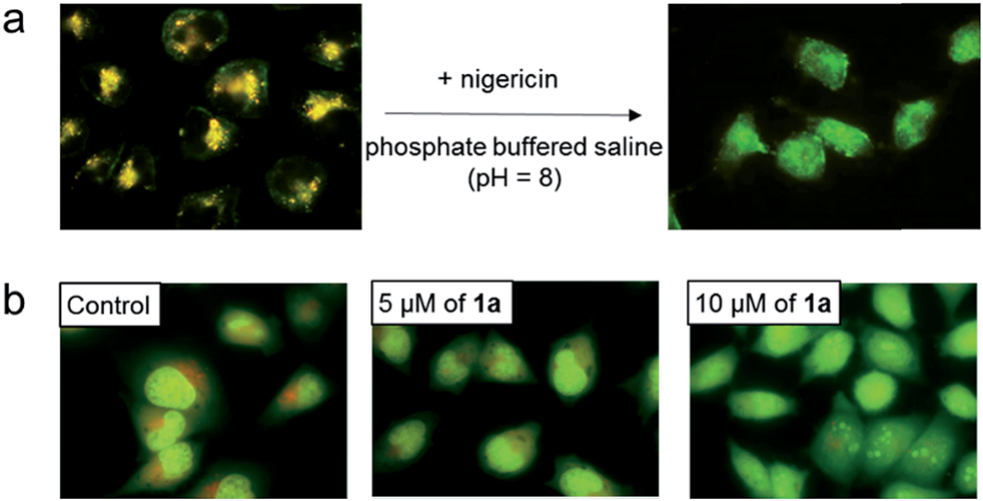
(a) Fluorescence images of HeLa cells after incubating with **2** for 1 h (left) and subsequently treated with nigericin in phosphate buffer (pH = 8). *E*_x_: 470 ± 20 nm, *E*_m_: >515 nm for both conditions. (b) Fluorescence microscopic analysis of HeLa cells treated with 5 or 10 μM of **1a** after 30 min at 37 °C and then stained with acridine orange. Results are typical of three independent experiments.

To further study the low pH-induced accumulation of Pt(ii) complexes in lysosomes, HeLa cells were incubated with nigericin, a H^+^/K^+^ ionophore that equalizes the pH across the lysosomes and cytosol to pH 8, in phosphate buffered saline (pH = 8), for 10 min after treatment of the cells with **2** for 1 h. As depicted in Fig. 7a, while a yellow-orange emission was detected upon treatment with **2** for 1 h, the emission color in cells with nigericin treatment turned green. The disappearance of the orange-yellow emission after equalizing the cellular pH to 8 further suggests that formation of the intracellular aggregates was induced in acidic compartments such as lysosomes in the cancer cells. Complexes **1a**, **1b**, **3**, **4**, and **5** all demonstrated similar intracellular localization (Fig. S12†).

We then tested whether accumulation of platinum(ii) complexes in lysosomes was associated with an increase in lysosomal membrane permeability (LMP). HeLa cells were first stained with acridine orange, a cell-permeable dye which gives red fluorescence in acidic lysosomes but exhibits green emission in the cytoplasm and nucleus. The cells were then treated with **1a** or **2** at different concentrations.^17^ In control cells, the red vesicles were clearly apparent (Fig. 7b and S13,† control). However, treatment of the cells with **1a** or **2** at 5 or 10 μM led to dose-dependent reductions of red fluorescence, indicative of increased LMP.^18^ Induction of LMP is a known cell death mechanism.^19^ Therefore, we examined the cytotoxicity of the platinum(ii) complexes towards different cancer cells by means of MTT (3-(4,5-dimethyl-2-thiazolyl)-2,5-diphenyltetrazolium bromide) (Table 1) and naphthol blue black (NBB) staining (Table S4†) assays. The complexes proved cytotoxic to cancer cells with IC_50_ values of 0.9–14.4 μM after 72 h treatment; these values were comparable to those of cisplatin (IC_50_ of 3.9–48.2 μM) under similar conditions. Complex **1a** (2 μM) also inhibited anchorage-independent colony formation in HeLa cells (Fig. S14†). *More importantly*, **1a***has been found to be cytotoxic towards primary bladder cancer cells collected from cancer patients*. As shown in Fig. S15,† **1a** showed cytotoxic IC_50_ value of 6.9 μM after 72 h incubation time, which was more potent than cisplatin (IC_50_ of ∼16 μM) under the same conditions.

**Table 1:** Cytotoxicity (IC_50_/μM, 72 h treatment) of the Pt(ii) complexes towards HeLa, breast cancer (MDA), ovarian carcinoma (A2780), nasopharyngeal carcinoma (SUNE1), B16 melanoma, hepatocellular carcinoma cells (HepG2) and non-tumorigenic immortalized liver cells (MIHA)^*a*^

Entry	HeLa	MDA	A2780	SUNE1	B16	HepG2	MIHA
**1a**	4.4 ± 0.6	1.6 ± 0.4	1.2 ± 0.1	3.5 ± 0.8	0.66 ± 0.04	1.3 ± 0.1	6.36 ± 0.15
**1b**	2.8 ± 0.2	2.1 ± 0.5	1.2 ± 0.1	2.8 ± 0.6	0.42 ± 0.02	1.7 ± 0.4	5.23 ± 0.62
**2**	5.1 ± 1.1	5.0 ± 0.2	3.3 ± 0.2	4.4 ± 1.0	0.85 ± 0.07	4.0 ± 0.8	8.12 ± 1.27
**3**	3.8 ± 0.5	4.2 ± 1.0	2.7 ± 0.1	3.9 ± 0.7	2.6 ± 0.2	2.7 ± 0.2	9.56 ± 2.15
**4**	8.3 ± 0.9	7.4 ± 0.5	5.0 ± 0.2	7.9 ± 1.3	3.0 ± 0.3	6.2 ± 0.6	13.51 ± 2.12
**5**	14.4 ± 3.5	8.3 ± 1.6	11.7 ± 2.1	12.3 ± 4.2	6.2 ± 0.5	9.0 ± 1.6	20.15 ± 2.92
Cisplatin	7.9 ± 1.2	25.0 ± 1.4	3.9 ± 0.8	9.7 ± 2.8	15.1 ± 0.9	48.2 ± 6.5	58.8 ± 6.1

^*a*^The IC_50_ values are determined by at least three independent assays.

### Sustained and pH-responsive release properties of **1a**-hydrogels

**Fig. 8:**
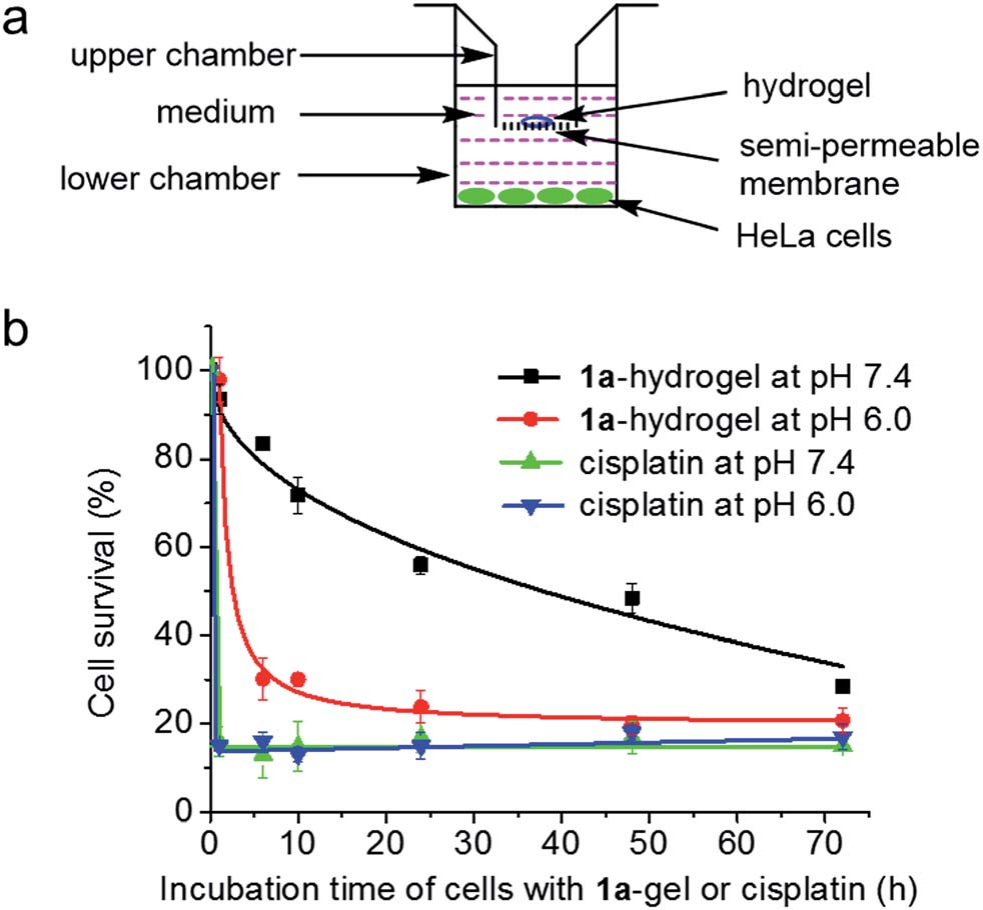
(a) Cytotoxicity and cell migration assay in Transwell® plates for sustained release experiments. (b) Time dependent survival of HeLa cells in the lower chamber with **1a**-hydrogel or cisplatin loaded in the upper chamber for different periods of time at pH 7.4 or 6.0. The total incubation time was 72 h.

We tested whether **1a**-hydrogel possesses sustained release properties in buffered solutions of varying pH (9, 7.4, 5 and 3) by UV/Vis absorption spectroscopy. As shown in Fig. S16,† **1a**-hydrogel showed time-dependent release activity at all pHs. It is noteworthy that **1a**-hydrogel showed much faster release activity at pH 5 and 3 compared to pH 7.4 and 9. The sustained cytotoxicity of **1a**-hydrogel towards HeLa cells was tested in double-chambered Transwell® 24-well plates (Fig. 8a). The plate has a semi-permeable membrane to separate the HeLa cells (lower chamber) from direct contact with hydrogel (upper chamber). After treatment of HeLa cells with **1a**-hydrogel (100 μM) at different time intervals, the upper chambers were removed and the HeLa cells were further incubated in the absence of the hydrogel. After a total of 72 h, the cell survival percentage was quantified by MTT assay. As shown in Fig. 8b, the cell survival gradually dropped from 1 h (93%), 6 h (83%), 10 h (72%), 24 h (56%), 48 h (48%), to 72 h (29%) at pH 7.4, suggesting a sustained release property. On the other hand, addition of a well-dispersed **1a** solution in the upper chamber (*i.e.*, **1a** is not in hydrogel form) caused the cell survival rate to rapidly drop to ∼15% after incubation for 1 h (Fig. S17†). Notably, if the pH of the cell culture medium was adjusted to 6.0, the cell survival decreased rapidly to ∼30% after a 6 h-treatment (Fig. 8b). On the other hand, the cells treated with cisplatin (100 μM; not present in a hydrogel form) under similar conditions rapidly died at both pH 7.4 and 6.0 (∼20% survival after a 1 h treatment; Fig. 8b). Therefore, an acidic environment can stimulate a faster release of **1a** from **1a**-hydrogel resulting in a more rapid cytotoxic effect. As the microenvironment of tumor tissues is often acidic compared to that of normal tissues,^20^**1a**-hydrogel could potentially achieve selective cytotoxicity towards tumor cells under *in vivo* conditions.

We also conducted an experiment to mimic intra-tumoral injection by seeding the cancerous HeLa cells in the upper chambers of Transwell® plates and a normal, non-tumorigenic immortalized liver cell line (MIHA) in the lower chambers, and then putting the **1a**-hydrogel in the upper chambers for 72 h.^10^ Only 13% of HeLa cells survived in the upper chambers while around 65% of the MIHA cells survived in the lower chambers. Thus, the sustained release property of **1a**-hydrogel could be used to modulate the cytotoxicity of **1a** by lessening the toxic side effect towards normal cells.

**Fig. 9:**
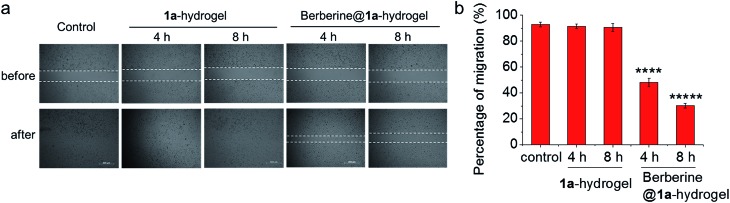
(a) Wound healing assay performed in Transwell® plates as in Fig. 8a. HeLa cells in the lower chamber were treated with **1a**-hydrogel or berberine@**1a**-hydrogel for 4 h and 8 h. Then the upper chamber was removed to allow the wound to recover. Photos were taken at 0 h and 24 h since the induction of wound. Figures are representative of three independent experiments. (b) Percentage of migration of HeLa cells after treatment with **1a**-hydrogel or berberine@**1a**-hydrogel. Data are shown as mean ± SEM from three independent experiments. ****p < 0.0001, *****p < 0.00001.

The ability of **1a**-hydrogel to carry other therapeutic drugs such as berberine was examined. Berberine is a natural product with extensive pharmacological applications; its potential anti-cancer properties, especially its anti-metastatic property is well documented.^21^ We found that in the presence of berberine, a viscous solution (denoted as berberine@**1a**-hydrogel) could also be formed after cooling a heated mixture of **1a** and berberine in water to room temperature (Fig. S18†). TEM analysis of berberine@**1a**-hydrogel showed partially aligned and entangled nanofibers of **1a** similar to **1a**-hydrogel (Fig. S19†). In phosphate buffered saline (pH 7.4), berberine was efficiently released from berberine@**1a**-hydrogel, as indicated by UV/Vis absorption experiments (Fig. S20†). We then tested whether the gel system could deliver berberine by performing cell migration experiments. As depicted in Fig. 9, HeLa cell mono-layers in the lower chamber of Transwell® plates with “wound” areas formed by scratching were treated with berberine@**1a**-hydrogel (berberine final concentration 8 μM) in the upper chamber for 4 h and 8 h. The wound of HeLa cells without treatment showed 93% recovery of the scratched area (Fig. 9a and b), while the treatment with berberine@**1a**-hydrogel significantly inhibited wound healing, showing only 48% and 30% of wound recovery after 4 h and 8 h treatments, respectively (Fig. 9a and b). In a comparative study, the cells treated with **1a**-hydrogel without berberine did not show inhibited cell migration with ∼91% of wound recovery after 4 h and 8 h treatments. Hence, **1a**-hydrogel could serve as a delivery vehicle for berberine to achieve anti-metastatic activity.

## Conclusion

In summary, we have developed a class of platinum(ii) complexes containing C-deprotonated pincer ligands with pyrazole groups, which display self-assembly and anti-cancer properties. These Pt(ii) complexes form aggregates in low-pH buffer solutions, accumulate in acidic lysosomes, increase lysosomal membrane permeability and exert cytotoxicity towards different immortalized cancer cells and a primary cancer cell. One of these complexes, **1a**, forms hydrogels in water, which displays sustained and pH-responsive release properties. The anti-cancer active **1a**-hydrogel could also be used to deliver berberine, and, in principle, other therapeutic agents to achieve dual therapeutic effects.

## Supplementary Material

Supplementary information

Crystal structure data
