# Supplementary material for: Luminescent platinum(ii) complexes with self-assembly and anti-cancer properties: hydrogel, pH dependent emission color and sustained-release properties under physiological conditions
Source: Chem Sci. 2015 Apr 28;6(7):3823–30. doi: 10.1039/c4sc03635b (PMC5707448; doi:10.1039/c4sc03635b)
Supplement: Supplementary file 1 [file SC-006-C4SC03635B-s001.pdf]

Electronic Supplementary Material (ESI) for Chemical Science.
This journal is © The Royal Society of Chemistry 2015

Electronic Supplementary Information

# Luminescent platinum(II) complexes with self-assembly and anti-cancer properties: hydrogel, pH dependent emission color and sustained–release properties under physiological conditions

Johnson Lui-Lui Tsai, Taotao Zou, Jia Liu, Tianfeng Chen, Anna On-Yee Chan, Chen Yang, Chun-Nam Lok and Chi-Ming Che*

# Contents

Materials and Methods S3

Experimental Procedures and Compound Characterization S4

Synthesis of ligand of **5** S4

Synthesis of **1a** S5

Synthesis of **1b** S5

Synthesis of **2** S6

Synthesis of **3** S6

Synthesis of **4** S6

Synthesis of **5** S6

X-ray Crystallographic Analysis S7

Formation of hydrogel from solution of **1a** S7

Sample preparation for electron microscopy S7

Biological Studies S7

Cell imaging S7

Co-localization study of **2** with Lysotracker®, Mitotracker®, or Hoechst 33342 S7

Preparation of tumor samples and primary cell culture S7

Cytotoxicity evaluation S8

Evaluating the cytotoxicity of **1a**-hydrogel S8

Microscopic visualization of lysosomes by acridine orange staining S9

Wound healing assay S10

Soft agar colony formation assay S10

Supporting Figures/Tables S11

References S41

## Materials and Methods

All chemicals, unless otherwise noted, were purchased from commercial sources. The sequence of the single strand DNA is 5'-CAATCCGGATTG-3' and the sequence of PNA is CAGTCCAGTT. All solvents for reaction and photophysical studies were of HPLC grade. $^1$H NMR spectra were recorded on a Bruker DPX 300, 400 or 500 M FT-NMR spectrometer with chemical shift (in ppm) relative to tetramethylsilane (for $CDCl_3$) or non-deuterated solvent residual. Absorption spectra were recorded on a Hewlett-Packard 8452A diode array spectrophotometer. Emission spectra were recorded on a SPEX Fluorolog-3 Model fluorescence spectrophotometer. Emission solutions for photophysical studies were degassed by using a high vacuum line in a two-compartment cell with at least five freeze-pump-thaw cycles. The emission quantum yield was measured with $[Ru(bpy)_3](PF_6)_2$ (Φ = 0.062) as reference. Emission lifetime measurements were performed with a Quanta Ray DCR-3 pulsed Nd:YAG laser system (pulse output 355 nm, 8 ns). Error limits were estimated: $\lambda$ (±1 nm); $\tau$ (±10 %); $\Phi$ (±10 %). Positive-ion mass spectra were recorded on a Finnigan MAT95 mass or Waters Micromass Q-Tof Premier quadrupole time-of-flight tandem mass spectrometer. Dynamic light scattering experiments were conducted using Zetasizer 3000HSA. Elemental analyses were performed by the Institute of Chemistry at the Chinese Academy of Sciences, Beijing.

Scanning electron microscopy (SEM) and transmission electron microscopy (TEM) experiments were taken on a Hitachi S-4800 FEG scanning electron microscope operating at 5.0 kV, and a Philips Tecnai G220 S-TWIN or CM 100 transmission electron microscope, respectively. All the samples for SEM observations were sputtered with gold thin film (10 second, <2 nm thickness).

Optical and fluorescence micrographs of cells were taken on a Zeiss Axiovert 200M inverted fluorescence microscopy. Confocal fluorescence micrographs were examined in a Carl Zeiss LSM510 META (Germany) laser scanning confocal microscope with a Plan Apochromat 63x 1.4NA oil immersion objective.

## Experimental procedure and compound characterization

All the isocyanide ligands (except 1-isocyano-4-(2-(2-methoxyethoxy) ethoxy)benzene, **E**) for syntheses of the Pt(II) complexes were from commercial sources, the synthetic route of **E** is as follows:

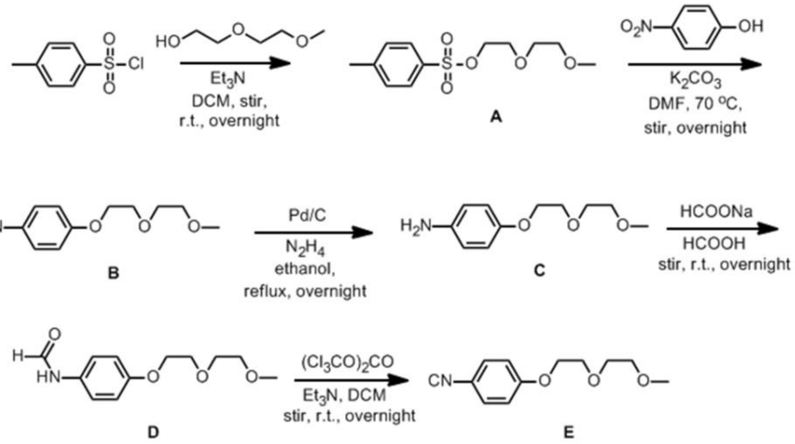


**A**: To a stirring solution of tosyl chloride (4 g, 0.021 mol) and diethylene glycol methyl ether (5.0 mL, 0.042 mol) in 30 mL of dried dichloromethane was added triethylamine (5.85 mL, 0.042 mol) at room temperature. After stirring for 2 h, the reaction mixture was extracted with 1 M $KHSO_4$ (aq) and 5% $NaHCO_3$ (aq). The organic layer was collected and evaporated. The crude product was then purified by column chromatograph. Yield: 5 g (86%). MS-EI(+): *m/z* 274.2 [$M^+$]. HRMS-EI(+) for $C_{12}H_{18}O_5S$, calcd. 274.0875, found 274.0870. $^1H$ NMR (400 MHz, $CDCl_3$, 298 K): $\delta$ = 7.80 (d, 2 H, *J* = 8.18 Hz), 7.34 (d, 2 H, *J* = 8.26 Hz), 4.17 (m, 2 H), 3.69 (m, 2 H), 3.59 (m, 2 H), 3.49 (m, 2 H), 3.35 (s, 3H), 2.45 (s, 3 H). **B**: A mixture of **A** (3 g, 0.011 mol), 4-nitrophenol (1.6 g 0.012 mol), and $K_2CO_3$ (3 g, 0.022 mol) in 15 mL of dimethylformamide was stirred at 70 $^oC$ overnight. Afterwards, the solvent was removed. The crude was re-dissolved in 25 mL ethyl acetate and extracted with 1 M NaOH (aq) and saturated NaCl (aq). The organic layer was collected and dried. Yield: 2.5 g (94%). MS-EI(+): *m/z* 241.1 [$M^+$]. HRMS-EI(+) for $C_{11}H_{15}NO_5$, calcd. 241.0950, found 241.0949. $^1H$ NMR (400 MHz, $CDCl_3$, 298 K): $\delta$ = 8.18 (d, 2 H, *J* = 10.52 Hz), 6.97 (d, 2 H, *J* = 10.54 Hz), 4.24 (m, 2 H), 3.91 (m, 2 H), 3.73 (m, 2 H), 3.58 (m, 2 H), 3.39 (s, 3H). **C**: To a mixture of **B** (1 g, 4.146 mmol) and 5% palladium on activated carbon (221 mg, 2.077 mmol) in 80 mL of ethanol was added hydrazine monohydrate (2.7 mL, 53.9 mmol). The reaction mixture was refluxed overnight. After cooling to room temperature, the solid was filtered and the filtrate was dried to give the desired product. Yield: 450 mg (51%). MS-EI(+): *m/z* 211.2 [$M^+$]. HRMS-EI(+) for $C_{11}H_{17}NO_3$, calcd. 211.1208, found 211.1200. $^1H$ NMR (400 MHz, $CDCl_3$, 298 K): $\delta$ = 6.75 (d, 2 H, *J* = 10.04 Hz), 6.63 (d, 2 H, *J* = 10.04 Hz), 4.07 (m,

2 H), 3.82 (m, 2 H), 3.71 (m, 2 H), 3.58 (m, 2 H), 3.39 (m, 5H). **E**: A mixture of **C** (300 mg, 1.422 mmol), formic acid (214.5 µL, 5.688 mmol), and sodium formate (19.3 mg, 0.284 mmol) was allowed to stir at room temperature overnight. 20 mL of dichloromethane was then added to the reaction mixture and the resultant mixture was extracted with $H_2O$ and saturated $NaHCO_3$ (aq). The solvent was removed from the organic layer to give product **D** which was used without further purification. To a solution of the product **D** (300 mg, 1.255 mmol) and triethylamine (500 µL, 3.766 mmol) in 5 mL of dichloromethane at 0 $^oC$ was added phosphoryl trichloride (175 µL, 1.882 mmol) dropwise through a syringe. The reaction mixture was then allowed to stir at room temperature overnight. 5 mL of saturated $Na_2CO_3$ solution was added to the reaction mixture and the resultant mixture was extracted with dichloromethane. The organic layer was collected and dried. The crude product was then purified by recrystallization from *n*-hexane. Yield: 170 mg (61%). MS-EI(+): *m/z* 221.1 [$M^+$]. HRMS-EI(+) for $C_{12}H_{15}NO_3$, calcd. 221.1054, found 221.1047. $^1H$ NMR (300 MHz, $CDCl_3$, 298 K): δ = 7.28 (d, 2 H, *J* = 9.01 Hz), 6.88 (d, 2 H, *J* = 8.95 Hz), 4.11 (m, 2 H), 3.85 (m, 2 H), 3.70 (m, 2 H), 3.57 (m, 2 H), 3.37 (s, 3H). Then the ligand was directly used for synthesis of the Pt(II) complex.

The platinum precursor [Pt(C^N^$N^{pyr}$)Cl] was synthesized according to the reported procedure.[1]

**1a**: The methodology for the previously reported isocyanide complexes[2] was adopted except that the substituted C^N^$N^{pyr}$ ligand was used. To a 10 mL $CH_3CN$ solution of [Pt(C^N^$N^{pyr}$)Cl] (100 mg, 0.222 mmol) was added 2,6-dimethylphenyl isocyanide (32 mg, 0.244 mmol). The reaction mixture was stirred at room temperature overnight. Then, diethyl ether was added to the reaction mixture. The crude product was collected by filtration and washed with diethyl ether. The product was recrystallized with $CH_3CN/Et_2O$. Yield: 90 mg (70%). MS-ESI(+): *m/z* 546 [M-Cl]$^+$. $^1H$ NMR (500 MHz, $CD_3OD$, 298 K): δ = 8.05 (m, 2 H), 7.76 (m, 2 H), 7.58 (m, 1 H), 7.36-7.11 (m, 6 H), 7.06 (m, 1 H), 2.50 (s, 6 H). Analysis (calcd., found for $C_{23}H_{19}ClN_4Pt \cdot 2H_2O$): C (44.70, 44.74), H (3.75, 3.60), N (9.07, 9.26).

**1b**: The methodology is similar to **1a** except with metathesis. Yield: 77%. MS-FAB(+): *m/z* 546 [M-OTf]$^+$. $^1H$ NMR (400 MHz, $CD_3CN$, 298 K): δ = 8.04 (t, 1 H, *J* = 8.16 Hz), 7.97 (d, 1 H, *J* = 2.71 Hz), 7.77-7.68 (m, 2 H), 7.58 (m, 1 H), 7.46 (m, 1 H), 7.40 (t, 1 H, *J* = 7.97 Hz), 7.30 (m, 2 H), 7.23-7.12 (m, 2 H), 7.02 (d, 1 H, *J* = 2.71 Hz), 2.55 (s, 6 H). Analysis (calcd., found for $C_{24}H_{19}F_3N_4O_3PtS \cdot 0.5H_2O$): C (40.91, 40.59), H (2.86, 2.59), N (7.95, 7.82).

**2**: The methodology is similar to **1a**. Yield: 75%. MS-ESI(+): *m/z* 498 [M-Cl]$^+$. $^1$H NMR (400 MHz, $d_6$-DMSO, 298 K): δ = 8.27 (m, 1 H), 8.31 (t, 1 H, *J* = 7.75 Hz), 7.88 (m, 2 H), 7.64 (m, 1 H), 7.26-7.07 (m, 4 H), 4.10 (m, 2 H), 1.86 (m, 2 H), 1.50 (m, 2 H), 1.00 (m, 3 H). Analysis (calcd., found for $C_{19}H_{19}ClN_4Pt$ • $H_2O$): C (41.35, 40.95), H (3.84, 3.51), N (10.15, 10.04).

**3**: The methodology is similar to **1a** except with metathesis. Yield: 85%. MS-FAB(+): *m/z* 548 [M-OTf]$^+$. $^1$H NMR (300 MHz, $CD_3CN$, 298 K): δ = 7.67 (d, 2 H, *J* = 5.63 Hz), 7.55 (t, 1 H, *J* = 7.57 Hz), 7.39 (m, 1 H), 7.20-6.71 (m, 8 H), 6.54 (m, 1 H), 3.89 (s, 3 H). Analysis (calcd., found for $C_{23}H_{17}F_3N_4O_4PtS$ • $4H_2O$): C (35.89, 35.61), H (3.27, 2.96), N (7.28, 7.32).

**4**: The methodology is similar to **1a** except with metathesis. Yield: 71%. MS-FAB(+): *m/z* 568 [M-OTf]$^+$. $^1$H NMR (300 MHz, $CD_3CN$, 298 K): δ = 8.29 (m, 1 H), 7.92-7.81 (m, 3 H), 7.75 (t, 1 H, J = 7.89 Hz), 7.61-7.55 (m, 3 H), 7.42-7.50 (m, 1 H), 7.38-7.29 (m, 2 H), 7.21 (d, 1 H, J = 7.12 Hz), 7.14 (d, 1 H, J = 8.16 Hz), 7.06-6.94 (m, 2 H), 6.68 (d, 1 H, J = 2.65 Hz). Analysis (calcd., found for $C_{26}H_{17}F_3N_4O_3PtS$ • $1.5H_2O$): C (41.93, 41.44), H (2.71, 2.32), N (7.53, 7.89).

**5**: The methodology is similar to **1a** except with metathesis. Yield: 65%. MS-ESI(+): *m/z* 636 [M-OTf]$^+$. $^1$H NMR (400 MHz, $CD_3CN$, 298 K): δ = 7.76-7.65 (m, 3 H), 7.58 (m, 1 H), 7.36-7.22 (m, 2 H), 7.18 (t, 1 H, *J* = 6.79 Hz), 7.05-6.93 (m, 4 H), 6.88 (m, 1 H), 6.69 (m, 1 H), 4.18 (t, 2 H, *J* = 6.60 Hz), 3.82 (t, 2 H, *J* = 4.22. Hz), 3.65 (t, 2 H, *J* = 4.86 Hz), 3.52 (t, 2 H, *J* = 3.64 Hz), 3.33 (s, 3 H). Analysis (calcd., found for $C_{27}H_{25}F_3N_4O_6PtS$ • $H_2O$ • $0.5CHCl_3$) : C (38.26, 38.20), H (3.21, 3.25), N (6.49, 6.67).

## X-ray Crystallographic Analysis

The procedure is similar to previous report.[3]

## Formation of hydrogel from solution of 1a

A suspension of **1a** in water (5% wt) was heated to 353 K affording a clear orange solution. The solution formed hydrogel after gradually cooling to 298 K.

## Sample preparation for electron microscopy

The samples for electron microscopy experiments were prepared by adding an ethanolic solution of the complexes onto a silicon wafer (SEM) or copper grid (TEM) and air-dried for 10 min. The samples were sputtered with gold thin film (10 s, $< 2$ nm thickness) before SEM analysis. The sample preparation of hydrogel was similar except air dried overnight.

## Biological studies

## Cell imaging

HeLa cells (2 x $10^5$) were grown on a 35 mm tissue culture dish with 2 mL culture medium and incubated at 37 °C under an atmosphere of 5% $CO_2$ for 24 h. The culture medium was removed, washed with PBS for 3 times, and incubated with medium containing the complexes at a concentration of 10 μM for 1 h. The medium was removed and washed with PBS for 3 times before imaging. For the time-dependent cell imaging experiment of **1a** or **2**, fluorescent images were taken at different incubation times without removing the old medium.

## Co-localization study of 2 with Lysotracker®, Mitotracker®, or Hoechst 33342

After incubating with 10 μM of **2** for 1 h, the HeLa cells were washed with PBS for 3 times and treated with medium containing LysoTracker® Red DND-99 (50 nM), MitoTracker (50 nM), or Hoechst 33342 (1 μM) at 37 °C under an atmosphere of 5% $CO_2$ for 30 min. The cells were then washed with PBS for 3 times and imaged using a Carl Zeiss LSM510 META (Germany) laser scanning confocal microscope.

## Preparation of tumor samples and primary cell culture

All these experiments were authorized according to Human Experimentation Ethics Committee of Jinan University. The specimens (weight at about 50 - 100 mg) were obtained at cystoscopy from a site 2 cm cephalic and 2 cm lateral to the left ureteric

orifice, stored in pre-cooled Hank's Balanced Solution (HBSS), and then cut with small scissor into about 2-3 $mm^3$. The specimens were then digested with 0.5% collagenase IV at 37 ℃ for 15 min, filtered through sterile 100 mesh filter screen. The cells were collected by centrifugation at 700 g for 5 min, and then re-suspended in DMEM culture medium supplemented with penicillin (100 units/ml), streptomycin (100 μg/ml) and fungazone (0.25 μg/ml) at 37 ℃ in 95% air, 5% $CO_2$, and cultured in 25 $cm^2$ culture flasks. The medium was replaced continuously every day. The interstitial cells were removed from the primary bladder cancer cells by trypsinization with 10-times diluted enzyme. The cells were used for drug screening when the confluence reached about 70%-80%. And the cell viability was examined by MTT assay.

## Cytotoxicity evaluation

**MTT assay** Cancer cells (cell density = 4000-6000 cells/100 μL) were seeded in a 96-well, flat-bottomed microtitre plate in growth medium (100 μL) and incubated at 37 ℃ under a 5% $CO_2$ atmosphere for 24 h. Different concentrations of complexes were added and the plates were incubated for 72 h. After a total incubation time of 72 h, MTT in PBS solution (5 mg/mL, 10 μL) was added to each well. The plates were incubated at 37 ℃ under a 5% $CO_2$ atmosphere for another 4 h, followed by adding 100 μL of sodium dodecyl sulfate (SDS, 10%) in diluted hydrochloric acid to each well. After further in dark at 37 ℃ overnight, the percentage of survival cells were determined with a microtitre plate reader at 590 nm.

**Naphthol blue black (NBB) staining assay** After a total incubation time of 72 h, the medium was removed and a mixture of 50 μL of formaldehyde (3% in PBS) and 50 μL of NBB solution (0.05% NBB, 0.1 M sodium acetate, 9% acetic acid) was added. After further 30 min incubation, the mixture was removed and each well was washed with deionized water for 3 times. The plates were air-dried and 100 μL of NaOH (50 mg/mL) was added to solubilize the NBB-stained cells. The plates were shaken for 1 h. The percentage of survival cells was determined with a microtitre plate reader at 590 nm.

## Evaluating the cytotoxicity of 1a-hydrogel[4]

HeLa cells (cell density = 4000 cells/100 μL) were seeded with 600 μL cultured medium in the lower chambers of the double-decked Transwell® 24-well plates and incubated at 37 ℃ under a 5% $CO_2$ atmosphere for 24 h. **1a**-hydrogel in 100 μL medium was then introduced to the upper chamber. The upper chambers were removed from the lower chamber at different time periods (1 h, 6 h, 10 h, 24 h, 48 h,

and 72 h). After a total incubation time of 72 h, MTT solution (5 mg/mL, 70 μL) was added to each well and the plate was further incubated for another 4 h followed by adding 700 μL of sodium dodecyl sulfate (SDS, 10%) in diluted hydrochloric acid to each well. After further in dark at 37 ℃ overnight, the percentage of survival cells were determined with a microtitre plate reader at 590 nm.

For the sustained cytotoxicity evaluation of **1a**-hydrogel at pH 6,[5] HeLa cells (cell density = 4000 cells/100 μL) were seeded with 600 μL cultured medium in the lower chambers of the double-decked Transwell® 24-well plates and incubated at 37 ℃ under a 5% $CO_2$ atmosphere for 24 h. The growth medium was then exchanged to pH 6.0 medium before adding **1a**-hydrogel in 100 μL pH 6.0 medium to the upper chamber. The upper chambers were detached from the lower chambers at different time periods (1 h, 6 h, 10 h, 24 h, 48 h, and 72 h). After a total incubation time of 72 h, the medium was changed back to growth medium and MTT solution (5 mg/mL, 70 μL) was added to each well. The plates were incubated at 37 ℃ under a 5% $CO_2$ atmosphere for another 4 h, followed by adding 700 μL of sodium dodecyl sulfate (SDS, 10%) in diluted hydrochloric acid to each well. After further in dark at 37 ℃ overnight, the percentages of survival cells were determined with a microtitre plate reader at 590 nm.

For mimicking the intratumoral injection experiment, HeLa cells were seeded in the upper chamber while the normal MIHA cells were seeded in the lower chamber for 24 h. After that, **1a**-hydrogel in 100 μL medium was introduced to the upper chamber. Further incubating for 72 h, MTT solution was added to both upper and lower chambers of each well and the plate was incubated for another 4 h followed by adding 700 μL of sodium dodecyl sulfate (SDS, 10%) in diluted hydrochloric acid to each well. After incubation in dark at 37 ℃ overnight, the percentage of survival cells were determined with a microtitre plate reader at 590 nm.

## Microscopic visualization of lysosomes by acridine orange staining

HeLa cells ($2 \times 10^5$) were seeded in a 35 mm tissue culture dish with 2 mL culture medium and grown at 37 $^oC$ under an atmosphere of 5% $CO_2$ for 24 h. The cells were incubated with 25 μM acridine orange at 37 $^oC$ under an atmosphere of 5% $CO_2$ for 15 min. After removing the medium and washing with PBS for 3 times, different concentrations of **1a** or **2** (5, 10, 25, 50, and 100 μM) were added and incubated at 37 ℃ under a 5% $CO_2$ atmosphere for 30 min.

## Wound healing assay

HeLa cells ($0.07 \times 10^6$) were seeded with 600 μL cultured medium in the lower chambers of the double-decked Transwell® 24-well plates and incubated at 37 ℃ under a 5% $CO_2$ atmosphere for 24 h. A uniform scratch was introduced on each well using a peptide tip and the images of the scratches were recorded (0 h). Berberine@**1a**-hydrogel and **1a**-hydrogel in 100 μL medium was separately introduced to the upper chamber. The upper chambers were detached from the lower chamber after 4 h or 8 h incubation time. The images of the scratches were captured after 24 h incubation time.

## Soft agar colony formation assay

HeLa cells ($1.5 \times 10^5$) were mixed with solvent control or **1a** (2 μM) in 2 mL medium with 0.35% soft agar per well in a 6-well plate coated with medium with 0.7% soft agar. 1 mL medium containing solvent control or **1a** (2 μM) was added to the soft agar after the soft agar was solidified. The imaging was captured after incubating the plate at 37 ℃ under a 5% $CO_2$ atmosphere for 7 day.

Supporting Figures/Tables

**Fig. S1** (a) $^1H$-$^1H$ COSY and (b) $^1H$-$^1H$ NOESY NMR spectra of **1a** in $d_4$-MeOH.

(a)

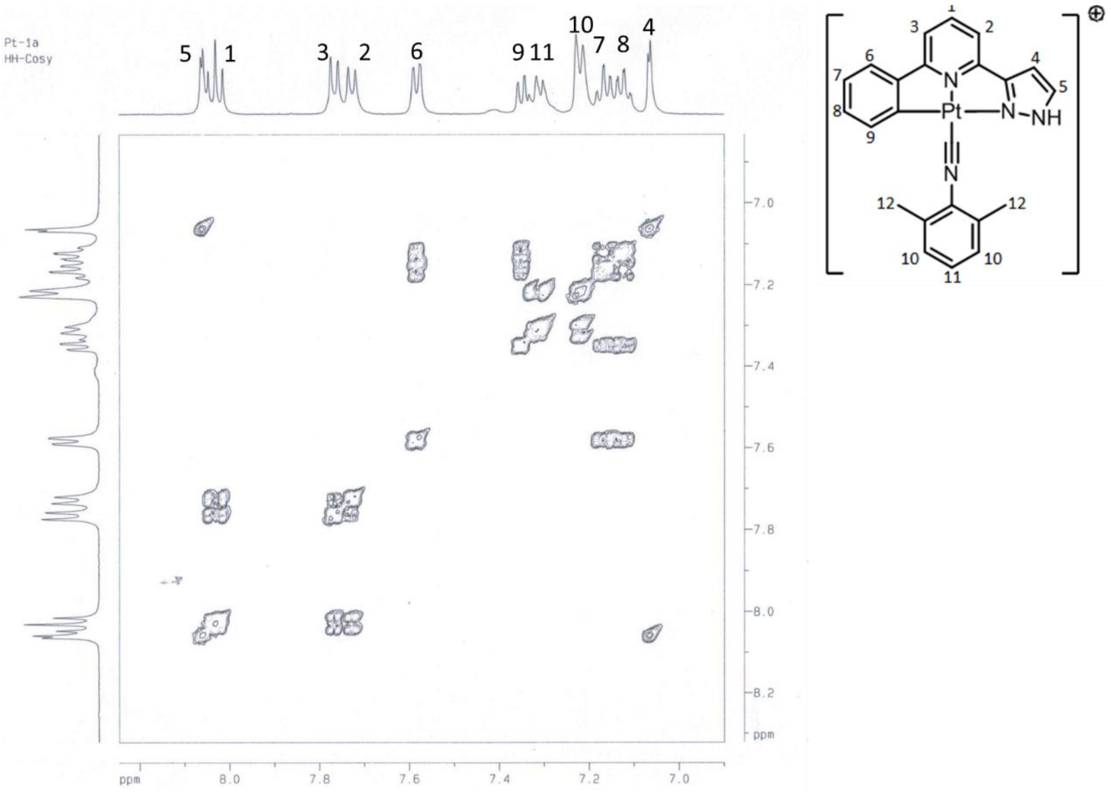


(b)

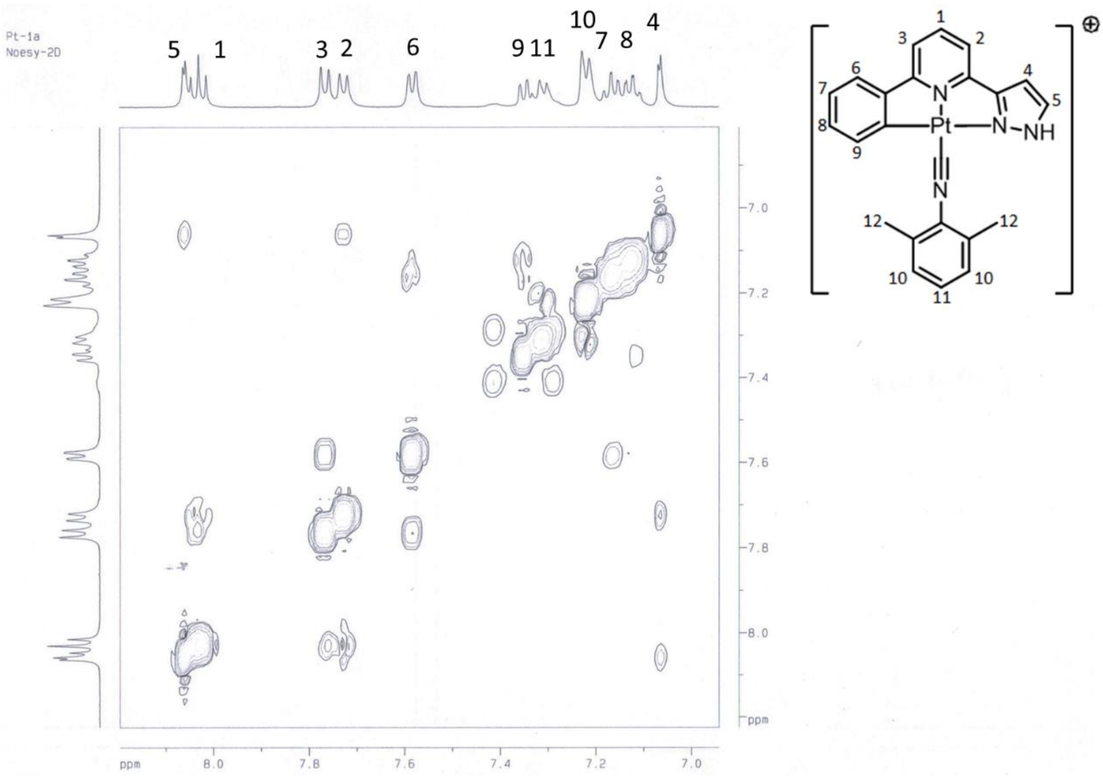

**Table S1**. Electronic absorption spectral data of the complexes at 298 K.

| Complex | Solvent | $\lambda_{abs}$/nm ($\varepsilon$/dm$^3$ mol$^{-1}$ cm$^{-1}$) |
|---|---|---|
| **1a** | $CH_2Cl_2$ | 248 sh (50,600), 315 (24,200), 333 sh (15,375), 346 (16,080) |
| | $CH_3CN$ | 246 (38,070), 310 (17,760), 329 sh (14,530), 342 (12,670) |
| | MeOH | 246 (24,980), 310 (10,840), 330 sh (8,640), 343 (7,380) |
| **1b** | $CH_2Cl_2$ | 259 sh (32,745), 313 (19,495), 334 sh (11,045), 347 (11,390) |
| | $CH_3CN$ | 242 (33,845), 265 sh (29,805), 308 (15,115), 329 sh (8,400), 343 (8,405) |
| | MeOH | 245 (21,540), 258 sh (19,530), 310 (9,700), 330 sh (6,945), 340 (6,225) |
| **2** | $CH_2Cl_2$ | 245 (31,090), 314 (15,410), 332 sh (9,740), 346 (9,930) |
| | $CH_3CN$ | 245 (17,550), 309 (8,825), 328 sh (7,250), 342 (6,530) |
| | MeOH | 245 (21,410), 314 (10,490), 329 sh (8,025), 343 (7,290) |
| **3** | $CH_2Cl_2$ | 262 sh (35,280), 312 (27,545), 334 sh (17,390), 347 (18,770) |
| | $CH_3CN$ | 259 (30,005), 308 (17,690), 330 sh (10,780), 342 (11,310) |
| | MeOH | 260 (23,000), 310 (13,890), 331 sh (10,245), 343 (9,965) |
| **4** | $CH_2Cl_2$ | 243 (74,960), 261 sh (51,775), 311 (35,180), 334 sh (21,465), 348 (23,370) |
| | $CH_3CN$ | 243 (56,545), 259 sh (39,670), 306 (24,170), 330 sh (14,080), 343 (14,610) |
| | MeOH | 242 (43,155), 260 sh (28,770), 308 (17,290), 331 sh (11,730), 344 (11,320) |
| **5** | $CH_2Cl_2$ | 262 sh (36,080), 313 (27,390), 334 sh (17,045), 347 (18,735) |
| | $CH_3CN$ | 260 (30,300), 309 (17,745), 330 sh (12,790), 343 (12,800) |
| | MeOH | 262 (29,215), 310 (17,460), 331 sh (13,140), 344 (12,720) |

**Fig. S2** Electronic absorption spectra of **1a** (a), **1b** (b), **2** (c), **3** (d), **4** (e), and **5** (f) in $CH_2Cl_2$, $CH_3CN$, and MeOH at 298 K.

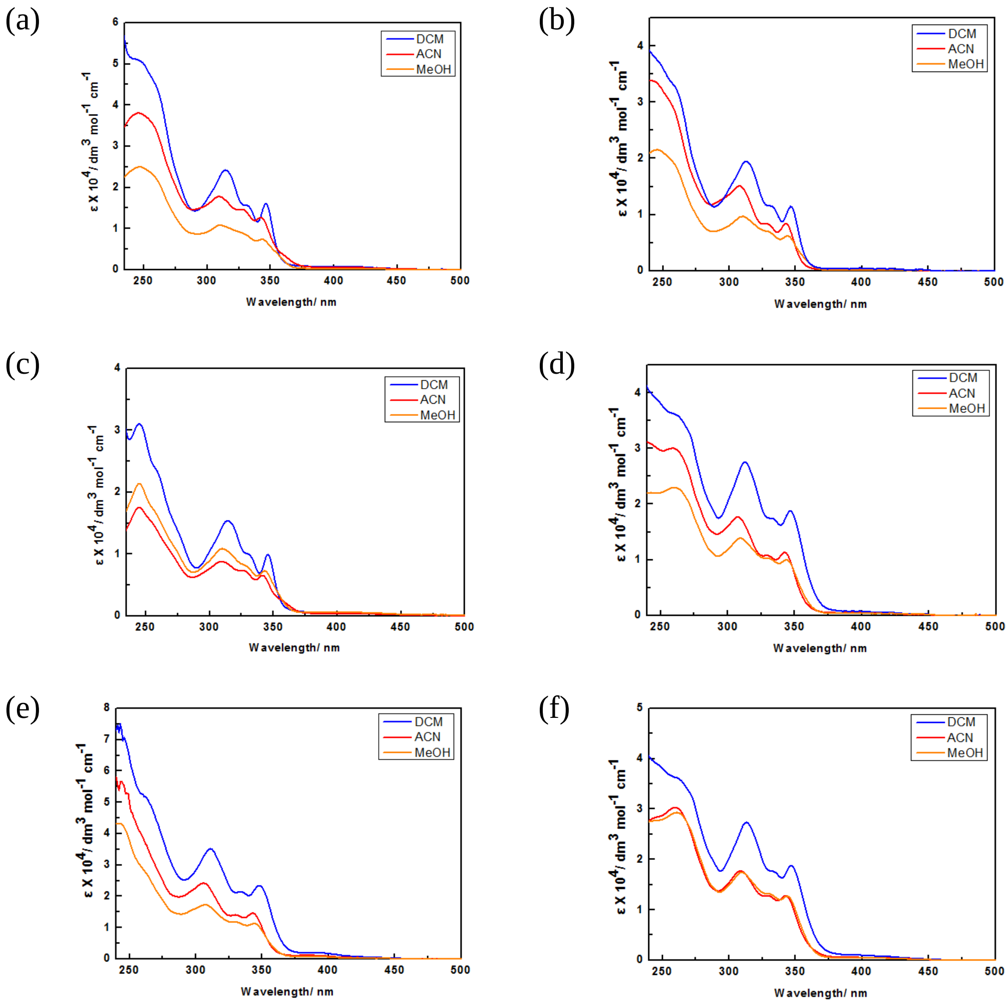

**Table S2.** Photophysical data of the complexes at 298 K.

| Complex | Medium | $\lambda_{em}$/nm | $\tau_o$/ μs | $\Phi_{em}$ |
|---|---|---|---|---|
| **1a** | $CH_2Cl_2$ | 503, 536, 580 sh | 11.6 | 9.8% |
| | $CH_3CN$ | 503, 536, 580 sh | 10.4 | 9.2% |
| | MeOH | 503, 538, 580 sh | 11.2 | 8.6% |
| **1b** | $CH_2Cl_2$ | 503, 536, 580 sh | 15.6 | 9.2% |
| | $CH_3CN$ | 503, 535, 580 sh | 9.10 | 6.8% |
| | MeOH | 503, 538, 577 sh | 10.1 | 8.2% |
| **2** | $CH_2Cl_2$ | 502, 536, 580 sh | 8.6 | 5.3% |
| | $CH_3CN$ | 502, 535, 580 sh | 7.4 | 6.4% |
| | MeOH | 502, 538, 579 sh | 8.9 | 10.4% |
| **3** | $CH_2Cl_2$ | 504, 537, 580 sh | 9.7 | 12.0% |
| | $CH_3CN$ | 505, 539, 585 sh | 11.9 | 13.6% |
| | MeOH | 503, 537, 580 sh | 11.8 | 9.8% |
| **4** | $CH_2Cl_2$ | 507, 536, 580 sh | 12.0 | 10.8% |
| | $CH_3CN$ | 507, 541, 582 sh | 9.0 | 11.3% |
| | MeOH | 504, 539, 581 sh | 11.5 | 10.2% |
| **5** | $CH_2Cl_2$ | 504, 536, 580 sh | 11.7 | 6.8% |
| | $CH_3CN$ | 505, 538, 584 sh | 10.9 | 6.0% |
| | MeOH | 503, 536, 580 sh | 12.1 | 19.6% |

**Fig. S3** Emission spectra of **1a** (a), **1b** (b), **2** (c), **3** (d), **4** (e), and **5** (f) in $CH_2Cl_2$ (DCM), $CH_3CN$ (ACN), and MeOH at 298 K.

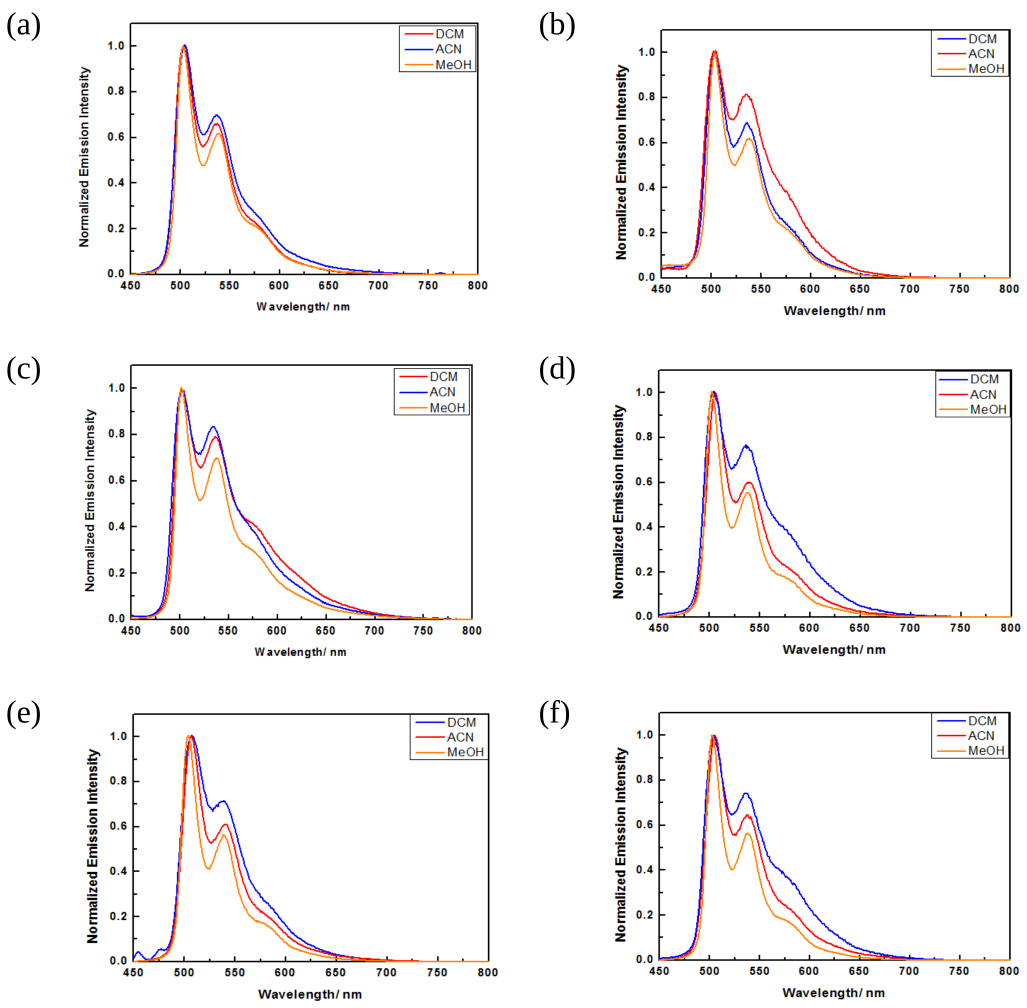

**Fig. S4** Absorption spectrum of **1a** in solutions of different $H_2O$:MeOH ratios at 298 K.

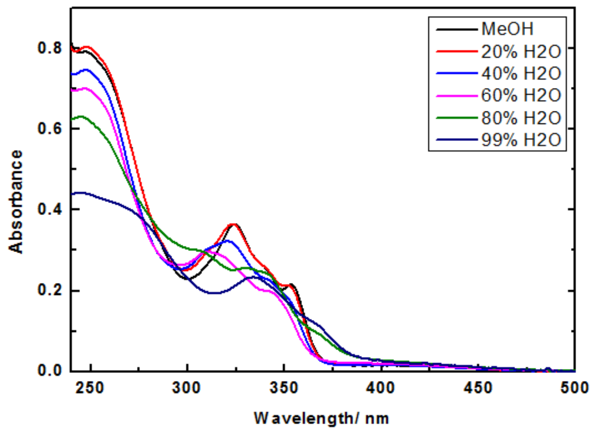

**Table S3.** Crystal data and structure refinement for complex **1**($ClO_4$).

| | |
|---|---|
| Identification code | **1**($ClO_4$) |
| Empirical formula | $C_{25}H_{21}ClN_5O_4Pt$ |
| Formula weight | 686.01 |
| Temperature/K | 100. |
| Crystal system | triclinic |
| Space group | P-1 |
| a/Å | 7.2307(3) |
| b/Å | 12.0600(5) |
| c/Å | 14.4710(6) |
| α/° | 74.4250(17) |
| β/° | 82.9797(18) |
| γ/° | 79.6363(18) |
| Volume/Å$^3$ | 1192.09(9) |
| Z | 2 |
| $\rho_{calc}$g/cm$^3$ | 1.911 |
| μ/mm$^{-1}$ | 12.412 |
| F(000) | 666.0 |
| Crystal size/mm$^3$ | 0.05 × 0.02 × 0.01 |
| Radiation | CuKα (λ = 1.54178) |
| 2Θ range for data collection/° | 6.36 to 134.444 |
| Index ranges | $-8 \leq h \leq 8$, $-14 \leq k \leq 14$, $-17 \leq l \leq 17$ |
| Reflections collected | 16896 |
| Independent reflections | 4132 [$R_{int}$ = 0.0724, $R_{sigma}$ = 0.0566] |
| Data/restraints/parameters | 4132/0/298 |
| Goodness-of-fit on F$^2$ | 1.068 |
| Final R indexes [I>=2σ (I)] | $R_1$ = 0.0404, $wR_2$ = 0.1075 |
| Final R indexes [all data] | $R_1$ = 0.0428, $wR_2$ = 0.1098 |
| Largest diff. peak/hole / e Å$^{-3}$ | 1.95/-1.50 |

**Fig. S5** SEM images of **1a** (a), **1b** (b), **3** (c), **4** (d), and **5** (e)

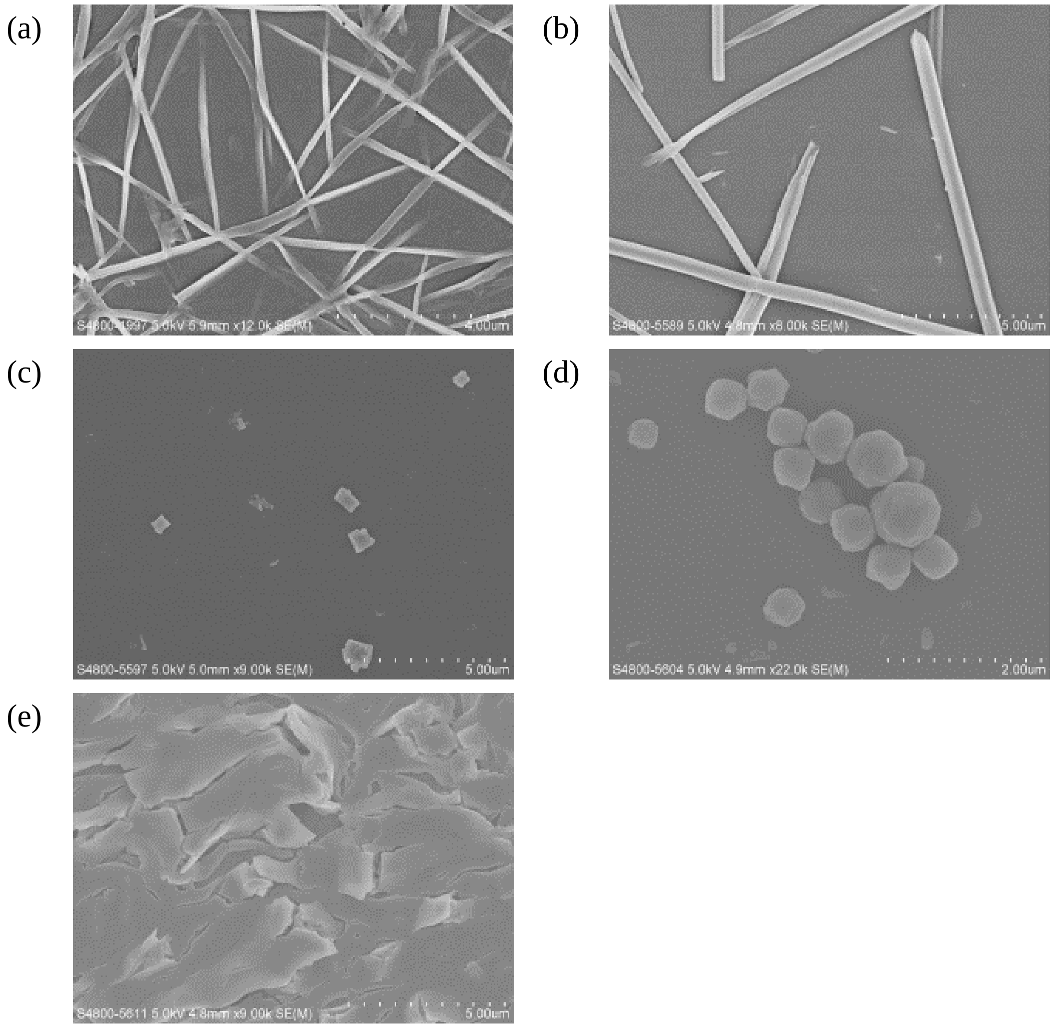

**Fig. S6** Dynamic light scattering experiment of **2** in PBS at pH = 4.

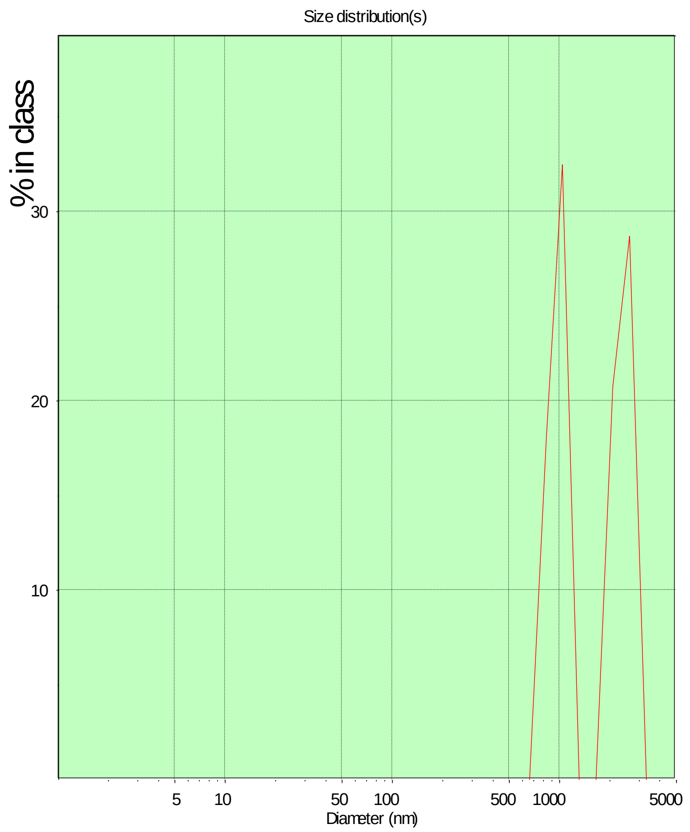


| Peak | Area | Mean | Width |
|---|---|---|---|
| 1 | 50.5 | 970.1 | 368.2 |
| 2 | 49.5 | 2403.8 | 1015.2 |

**Fig. S7** Emission spectra of **1a** (a), **1b** (b), **3** (c), **4** (d), and **5** (e) at different pH in 1% DMSO in phosphate buffer.

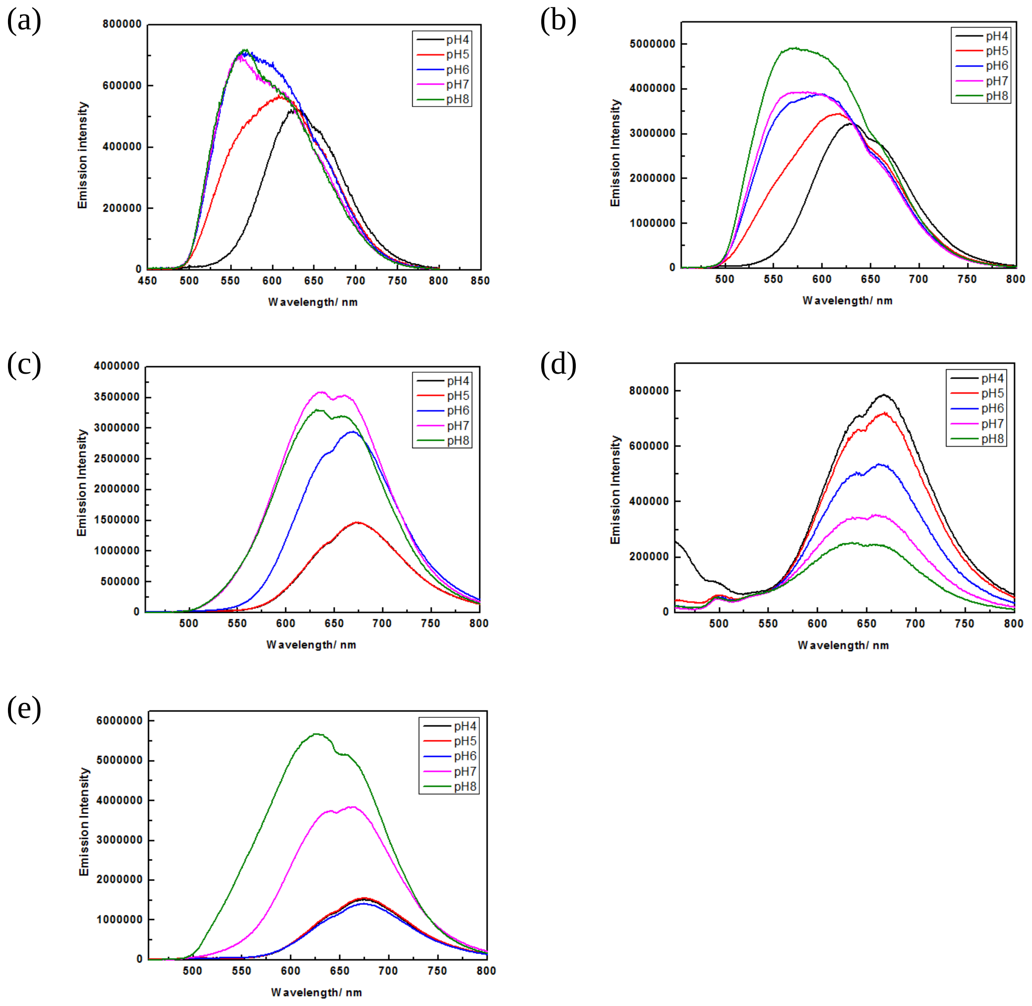

**Fig. S8** The emission spectra of **1a** (top) and **1b** (down) in the presence or absence of equal molar concentration of ssDNA or PNA.

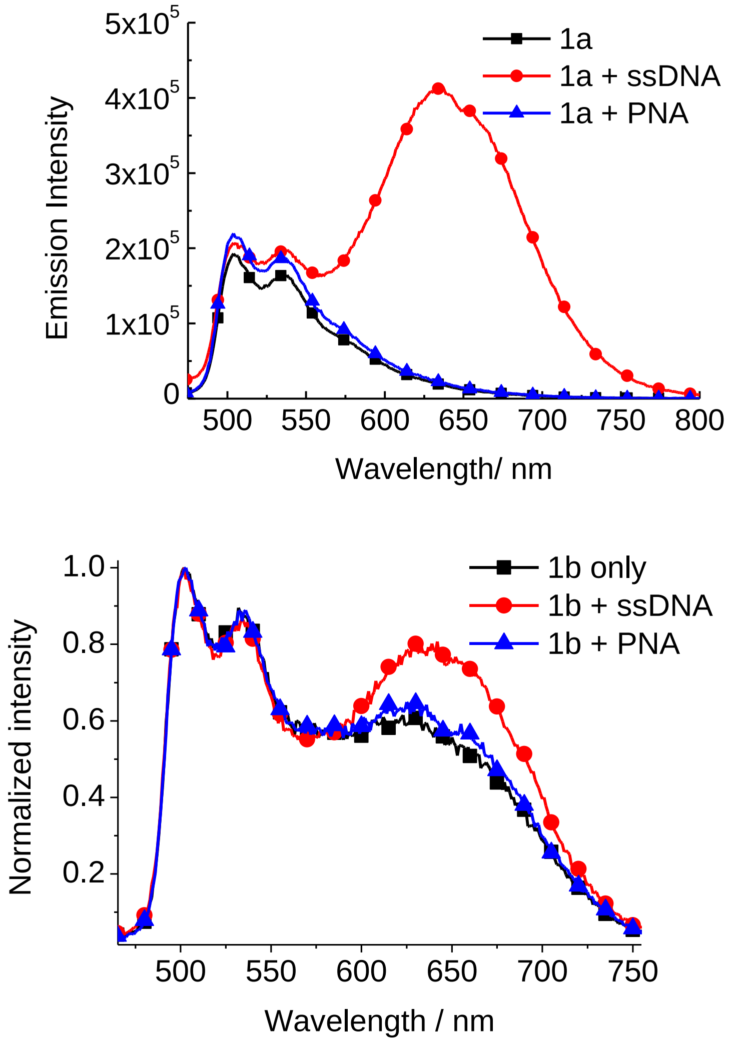

**Fig. S9** The emission spectra of **1b** in the presence of ssDNA at pH from 2.8 to 8.3.

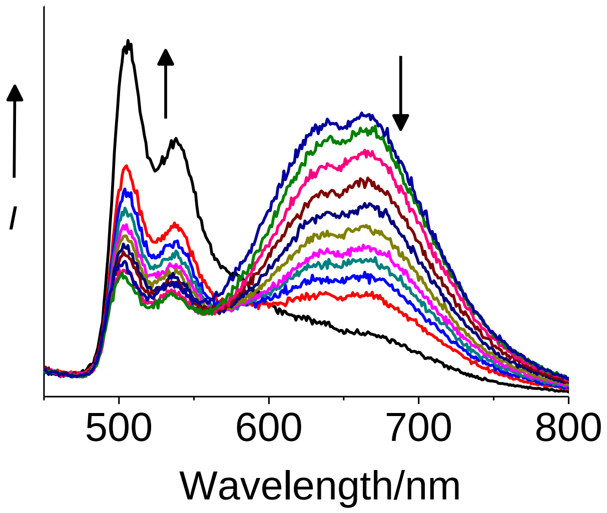

**Fig. S10** Fluorescence images of HeLa cells incubated with 10 μM of a) **1a** or b) **2** for different time periods.

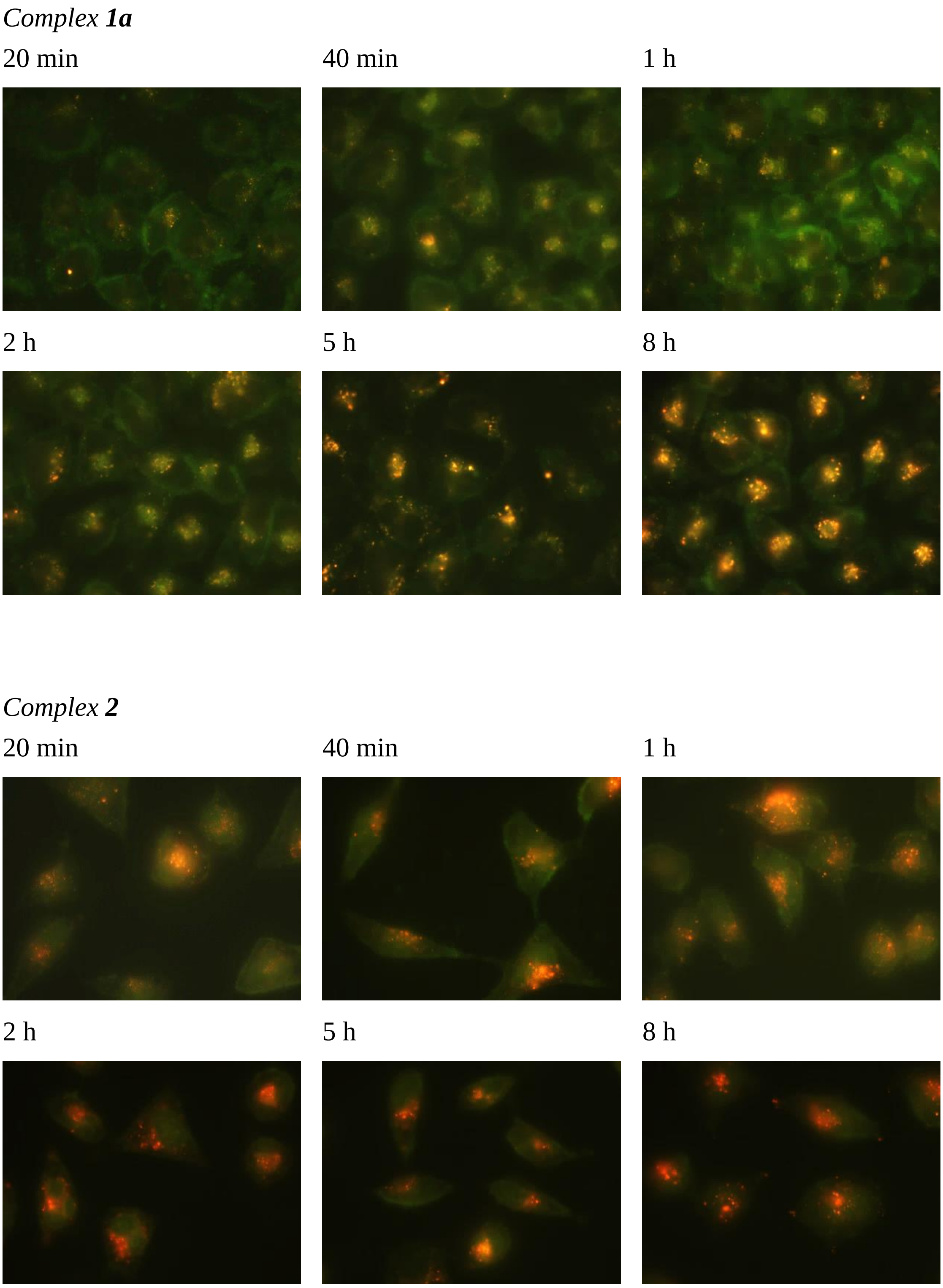

**Fig. S11** Fluorescence microscopic analysis of HeLa cells incubated with **2** (10 μM, 37 $^{o}$C, 1 h).

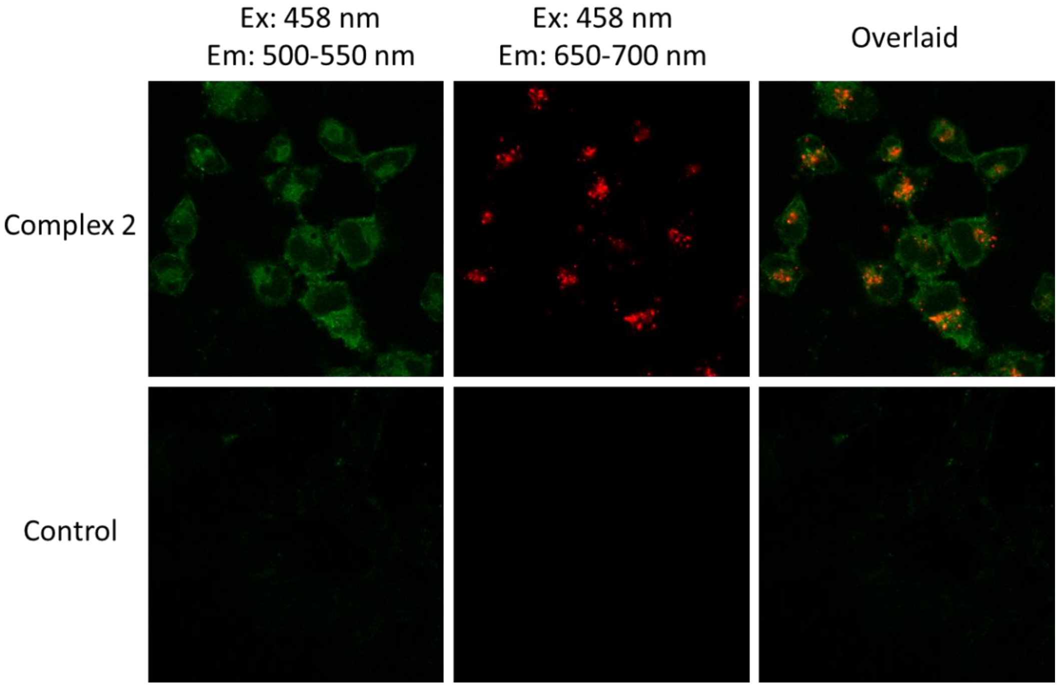

**Fig. S12** Fluorescence images of HeLa cells incubated with **1a** (a), **1b** (b), **2** (c), **3** (d), **4** (e), and **5** (f), respectively (10 μM, 37 °C, 1 h).

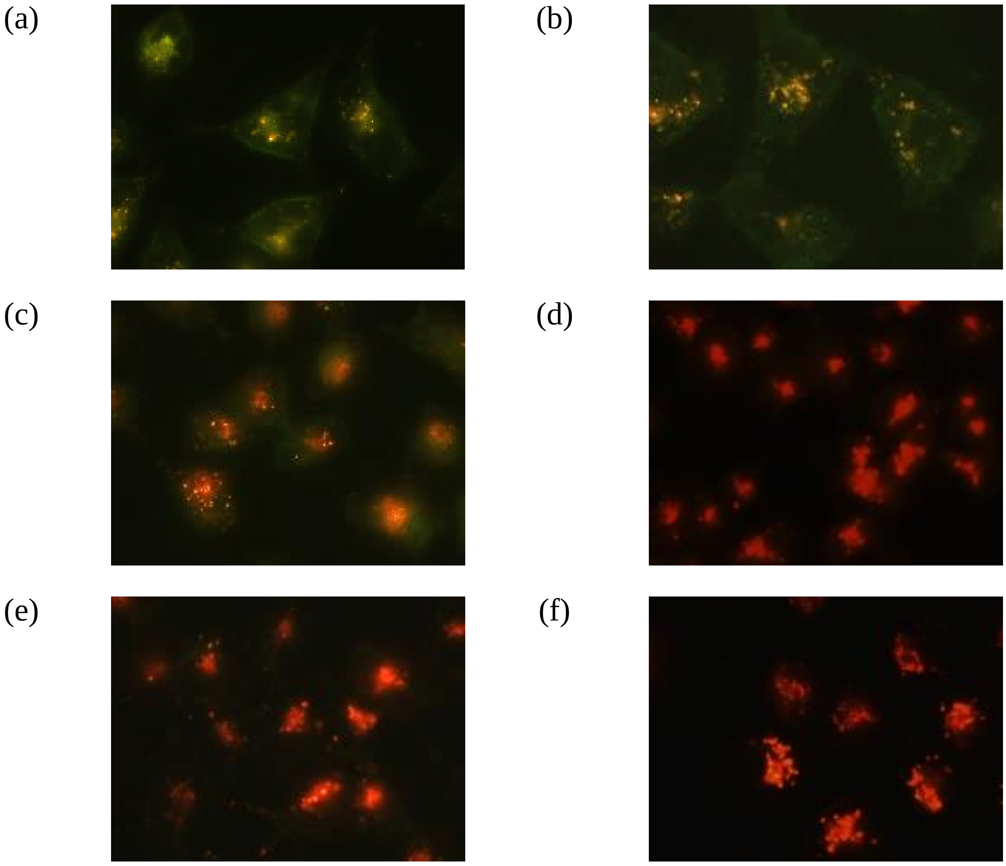

**Fig. S13** Fluorescence microscopic analysis of HeLa cells stained with acridine orange followed by incubation with different concentrations of **2** for 30 min at 37 $^{o}$C.

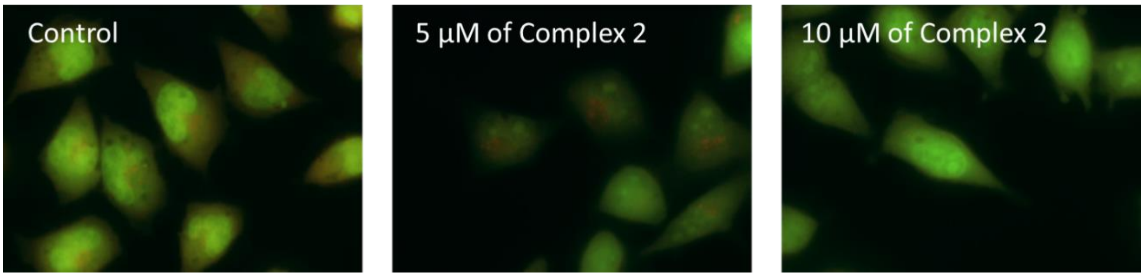

**Table S4.** Anti-proliferative activities of Pt(II) complexes towards HeLa cells as determined by naphthol blue black (NBB) staining assay.

| **1a** | **1b** | **2** | **3** | **4** | **5** | cisplatin |
|---|---|---|---|---|---|---|
| 1.05±0.06 | 0.90±0.17 | 2.28±0.18 | 1.98±0.21 | 3.52±0.89 | 9.47±1.92 | 4.29±0.36 |

**Fig. S14** Soft agar colony formation assay of HeLa cells in the presence of **1a**. Pictures shown were taken at 50× magnification. Representative pictures from three independent experiments.

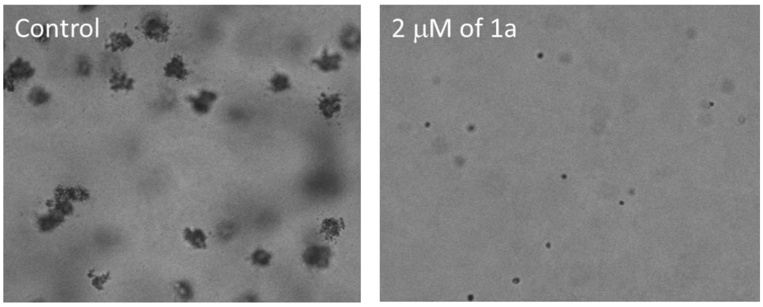

**Fig. S15** Cytotoxic MTT assay of the Pt complex towards primary bladder cancer cells.

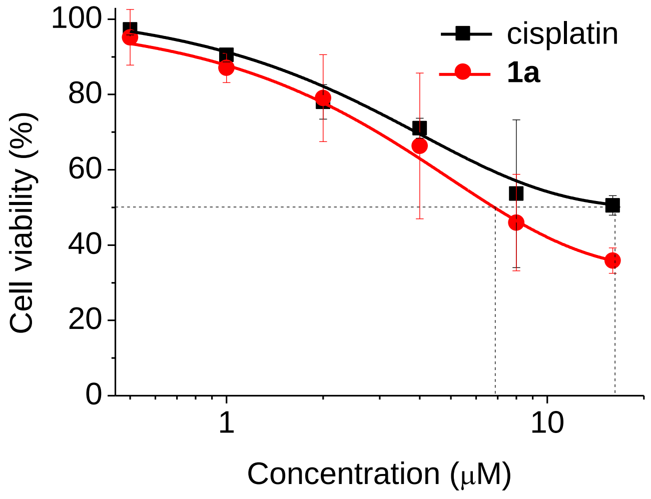

**Fig. S16** Electronic absorption spectra of **1a**-hydrogel showing its sustained release property in pH 3.0 (a), pH 5.0 (b), pH 7.4 (c) and pH 9.0 (d) PBS solution (containing 10% MeOH) at 298 K.

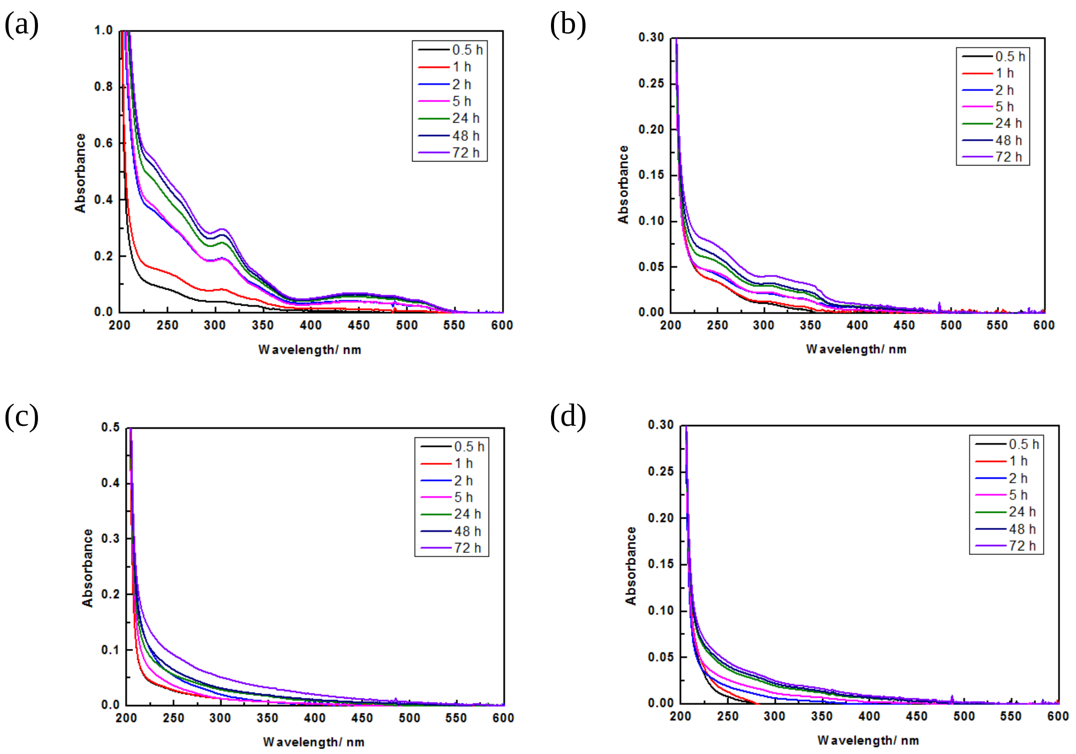

**Fig. S17** Time dependent survival of HeLa cells in the lower chamber of Transwell® plates with **1a**-hydrogel or cisplatin loaded in the upper chamber for different periods of time at pH 7.4. The total incubation time was 72 h.

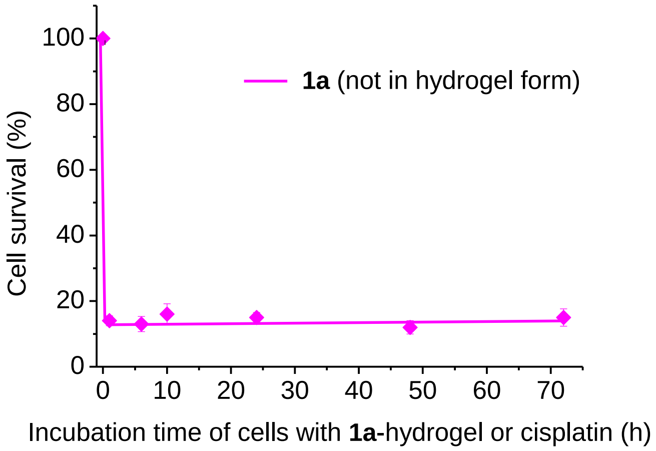

**Fig. S18** Images showing (a) addition of berberine in DMSO to suspension of **1a** at 80 °C and (b) formation of berberine@**1a**-hydrogel after cooling the solution to room temperature.

(a)

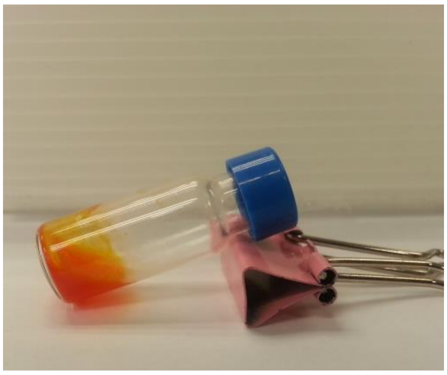

(b)

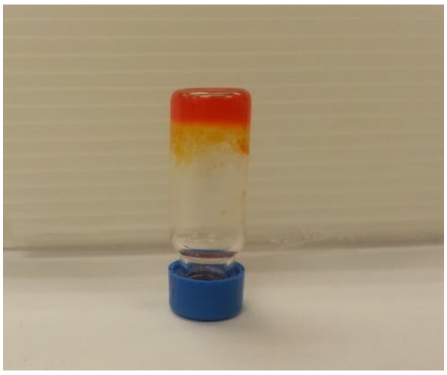

**Fig. S19** TEM image of berberine@**1a**-hydrogel.

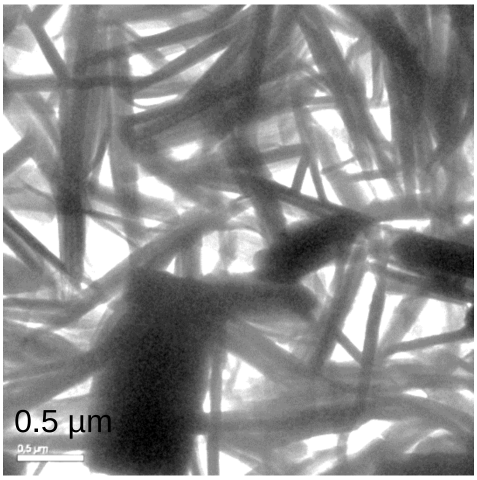

**Fig. S20** The release of berberine from berberine@**1a**-hydrogel (a) and berberine only (b) in pH 7.4 PBS solution at 298 K as shown by UV-Vis spectrophotometry.

a)

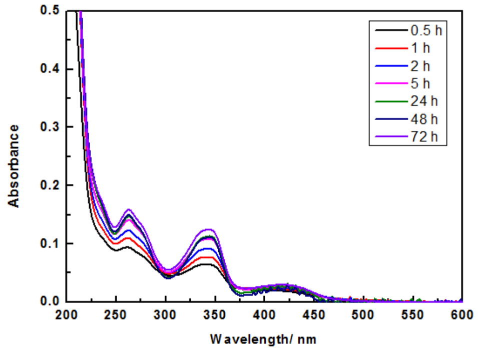


b)

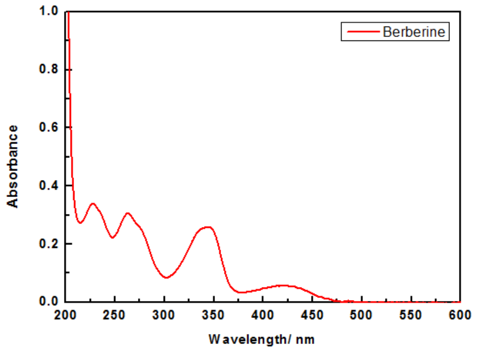

1a

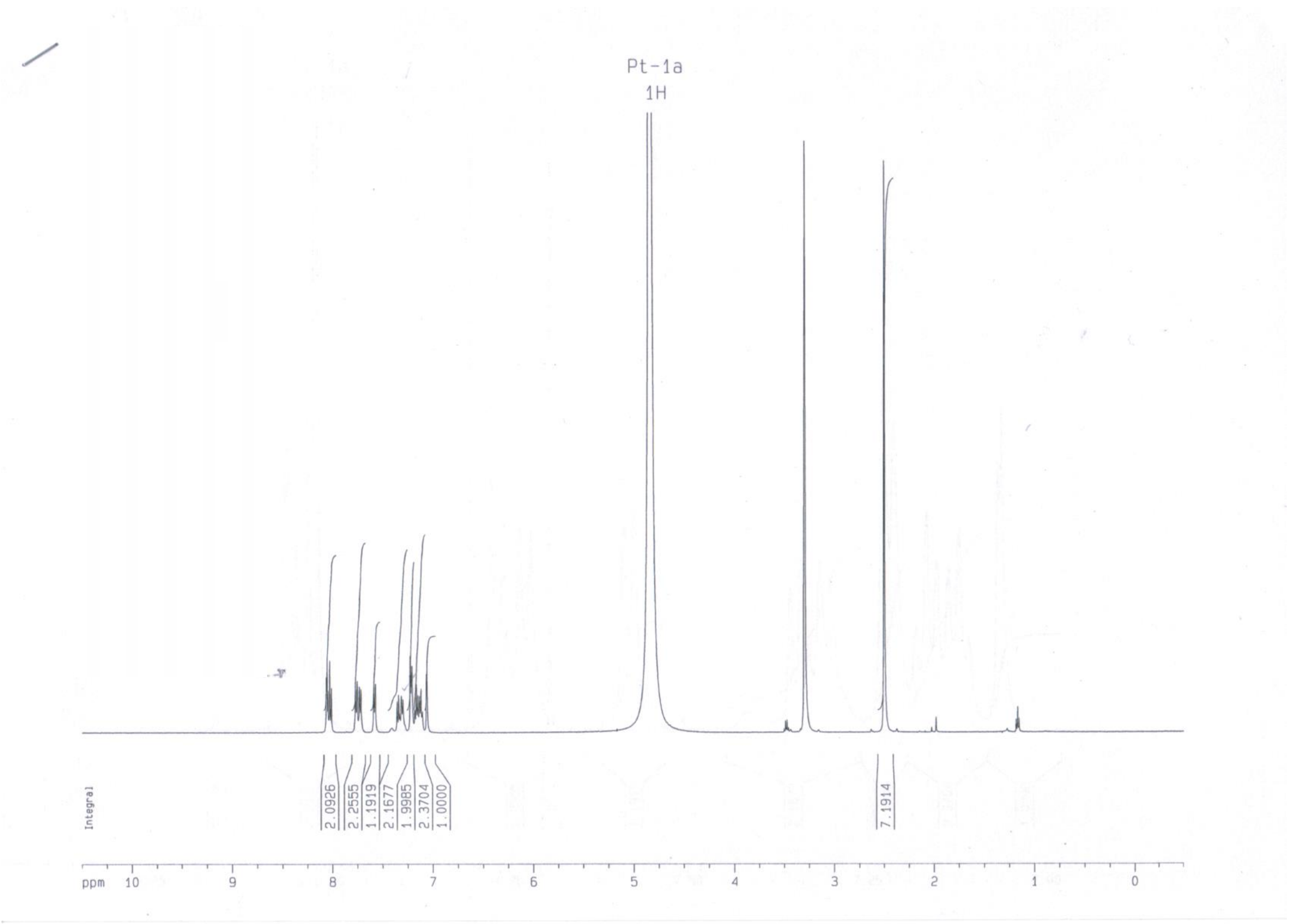

Pt-1a
1H
2.0926
2.2555
1.1919
2.1677
1.9985
2.3704
1.0000
7.1914
Integral
ppm 10 9 8 7 6 5 4 3 2 1 0

1b

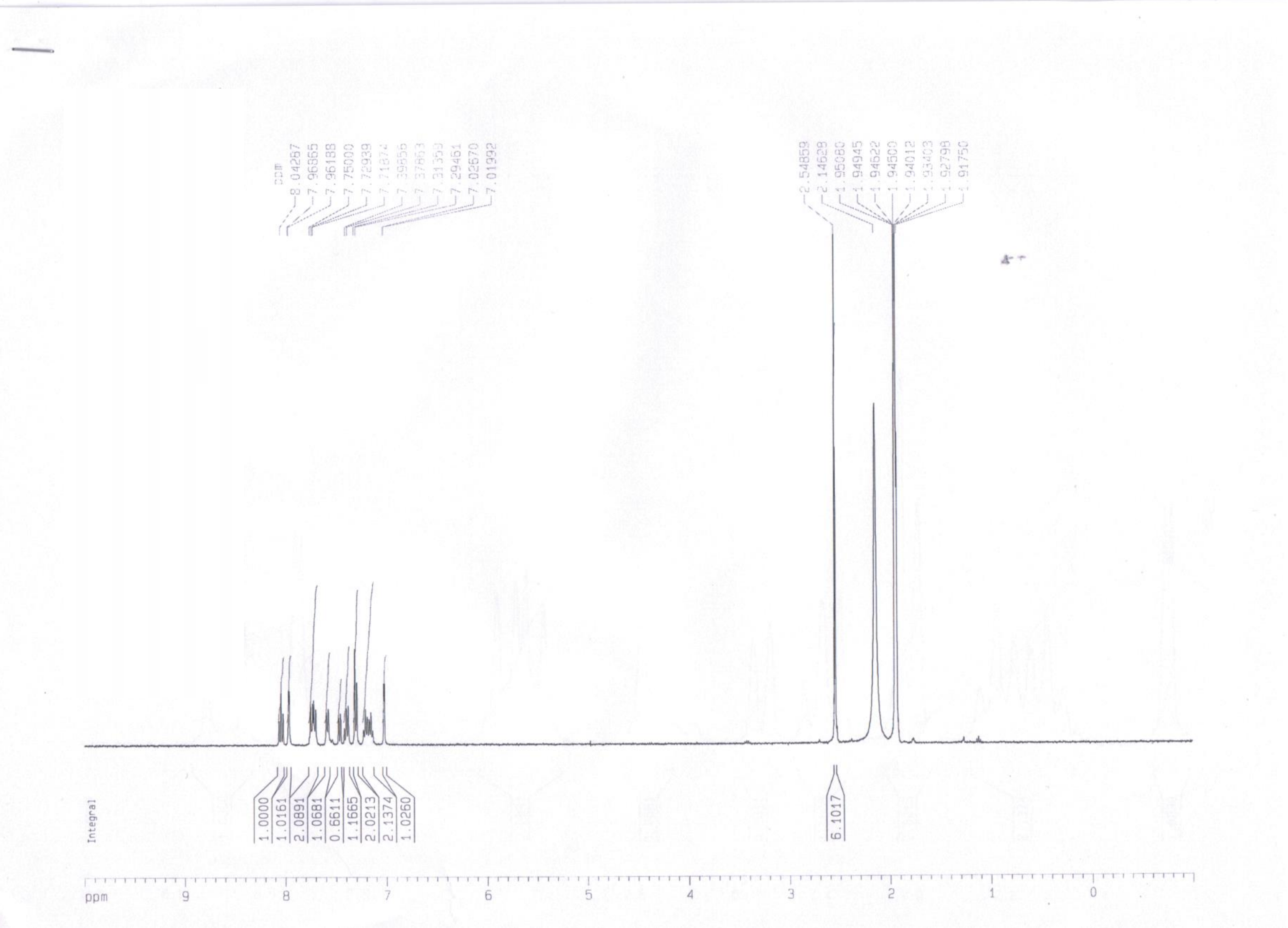

2

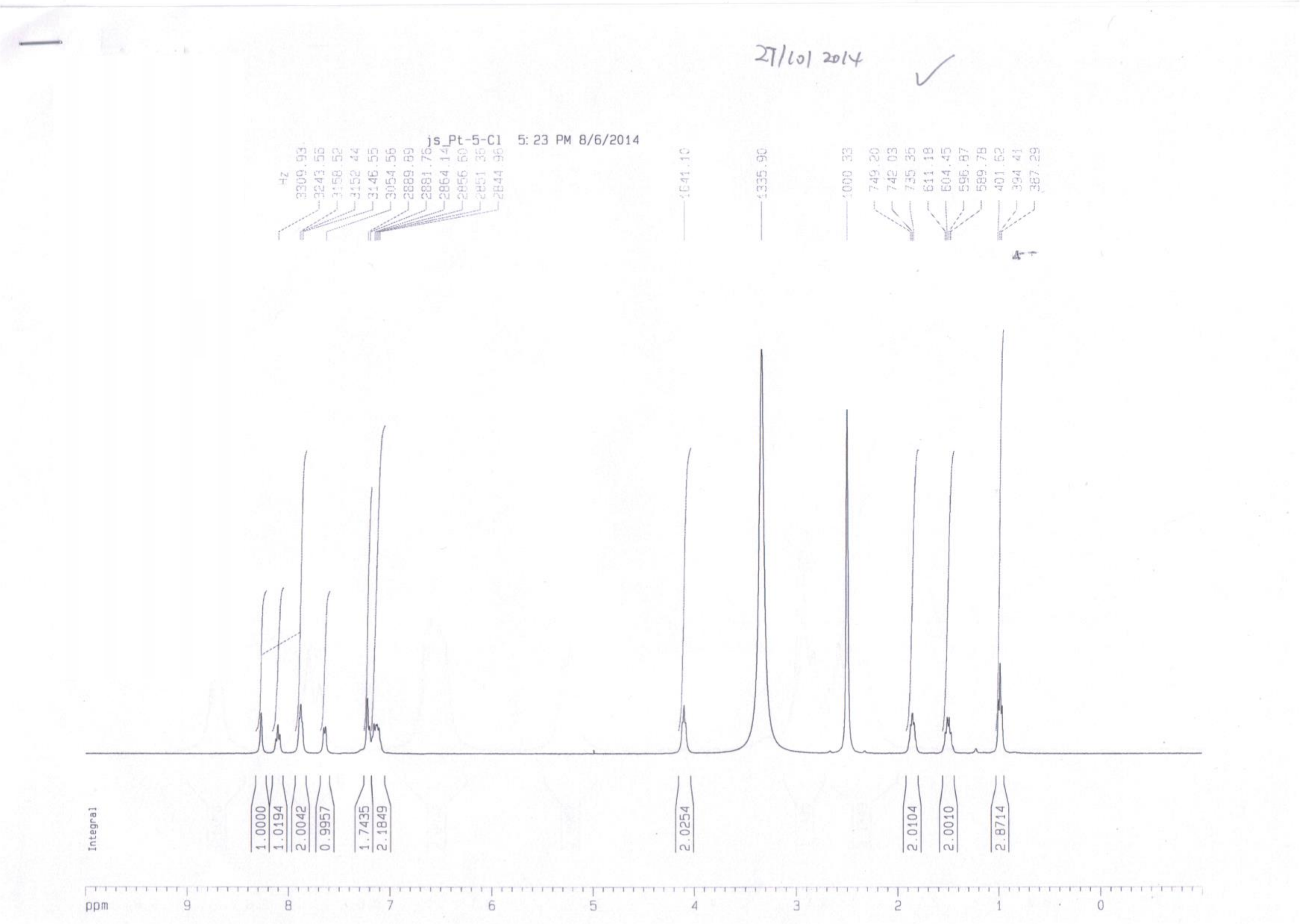

3

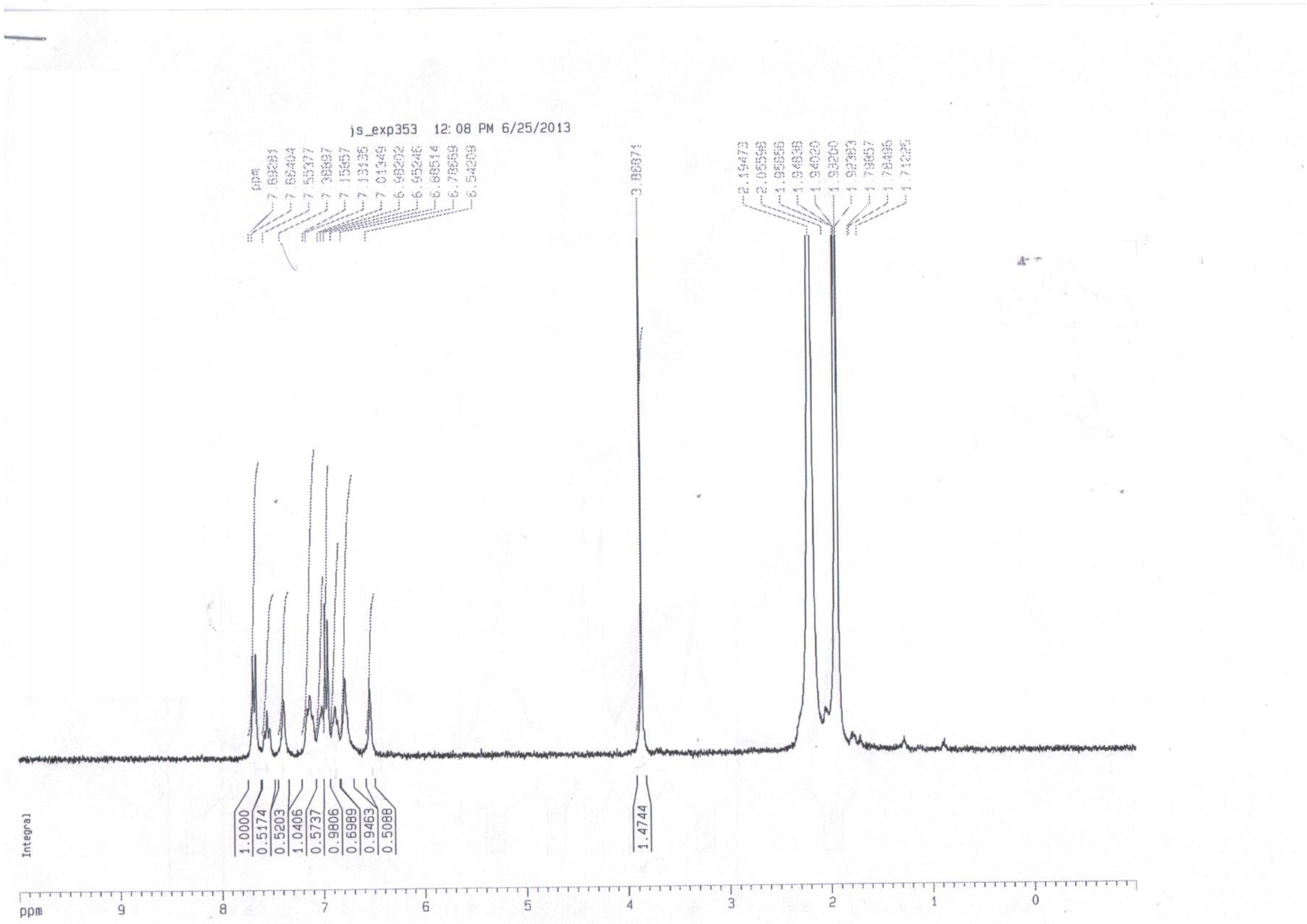

4

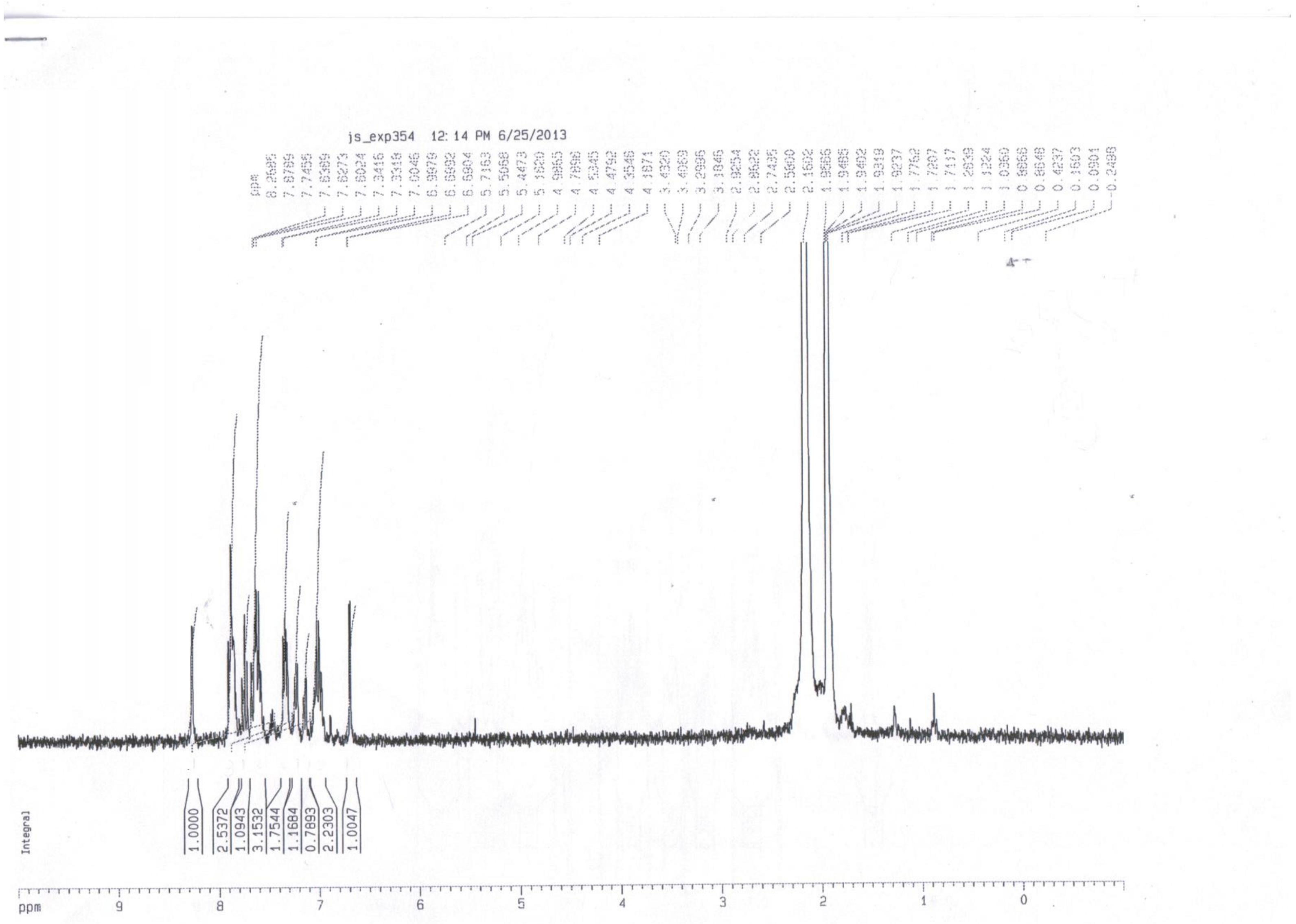

5

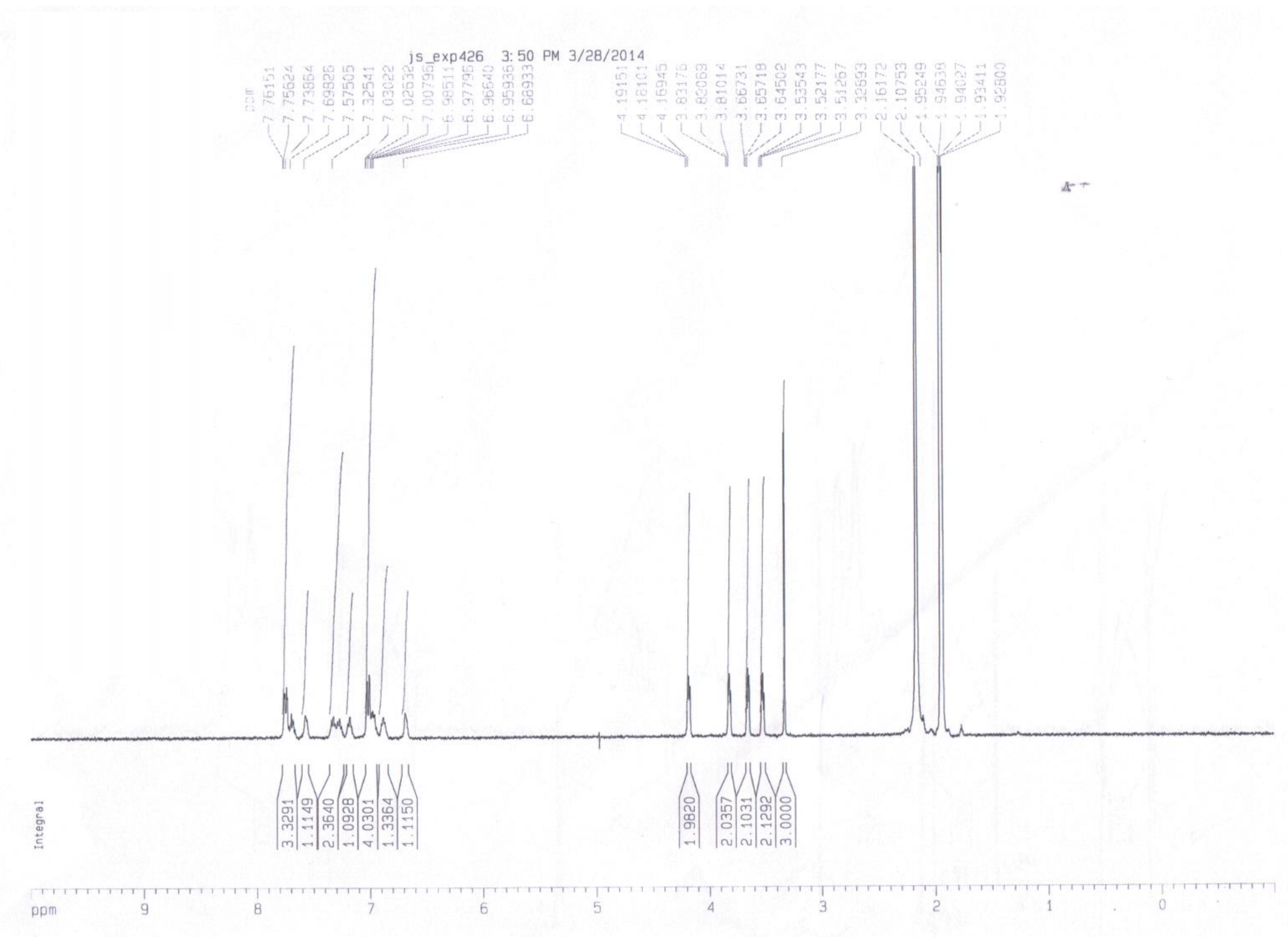

## References


[1] C.-K. Koo, B. Lam, S.-K. Leung, M. H.-W. Lam and W.-Y. Wong, *J. Am. Chem. Soc.*, 2006, **128**, 16434-16435.

[2] (a) S.-W. Lai, M. C.-W. Chan, Y. Wang, H.-W. Lam, S.-M. Peng and C.-M. Che, *J. Organomet. Chem.*, 2001, **617**, 133-140; (b) S.-W. Lai, M. C.-W. Chan, K.-K. Cheung and C.-M. Che, *Organometallics*, 1999, **18**, 3327-3336.

[3] (a) T. Zou, C. T. Lum, C. -N. Lok, W.-P. To, K.-H. Low and C.-M. Che, *Angew. Chem. Int. Ed.*, 2012, **51**, 4882-4886; (b) G. M. Sheldrick, Acta Cryst., 2008, **A64**, 112-122.

[4] J.-J. Zhang, W. Lu, R. W.-Y. Sun and C.-M. Che, *Angew. Chem. Int. Ed.*, 2012, **51**, 4882-4886.

[5] T. J. Moyer, J. A. Finbloom, F. Chen, D. J. Toft, V. L. Cryns and S. I. Stupp, *J. Am. Chem. Soc.*, 2014, **136**, 14746-14752.
